# The Association between Nonsuicidal Self-Injury and Perfectionism in Adolescence: The Role of Mental Disorders

**DOI:** 10.3390/ejihpe13110163

**Published:** 2023-10-24

**Authors:** Dora Gyori, Bernadett Frida Farkas, Daniel Komaromy, Lili Olga Horvath, Nora Kollarovics, Peter Garas, Judit Balazs

**Affiliations:** 1 Doctoral School of Psychology, Eotvos Lorand University, 1075 Budapest, Hungary; 2 Institute of Psychology, Eotvos Lorand University, 1075 Budapest, Hungary; d.komaromy@uva.nl (D.K.); horvath.lili@ppk.elte.hu (L.O.H.); 3 Mental Health Sciences Doctoral School, Semmelweis University Doctoral School, 1083 Budapest, Hungary; farkas.bernadett@phd.semmelweis.hu (B.F.F.); kollarovics.nora@phd.semmelweis.hu (N.K.); garas.peter@phd.semmelweis.hu (P.G.); 4 Department of Behavioural and Movement Sciences, Vrije Universiteit, 1081 HV Amsterdam, The Netherlands; 5 Pedagogical Services, 1141 Budapest, Hungary; 6 Department of Psychology, Oslo New University College, 0456 Oslo, Norway

**Keywords:** nonsuicidal self-injury, NSSI, NSSI functions, perfectionism, maladaptive perfectionism, adolescence

## Abstract

Previous evidence has drawn attention to the fact that maladaptive perfectionism is a risk factor for engagement in nonsuicidal self-injury (NSSI). Until now, few studies have examined this topic, especially among community adolescents. The aim of this study was to explore the relationship between perfectionism dimensions and NSSI functions to examine the potential mediating effect of mental disorders. Altogether, 146 Hungarian community adolescents (ages 13–18 years) were involved. All participants completed the Hungarian adaptation of the Inventory of Statements about Self-Injury (ISAS), the Frost Multidimensional Perfectionism Scale (FMPS), and the Mini International Neuropsychiatric Interview Kid. To analyse the interrelationships among NSSI, perfectionism, and mental disorders, we conducted regression and network analysis. Of the 146 adolescents, 90 (61.64%, girls: 71.11%) engaged in NSSI. The Concern over Mistakes and Doubts about Action scales of the FMPS significantly and positively predicted both NSSI intrapersonal and interpersonal motivation, with comparable effect sizes, and this association was fully mediated by anxiety disorders. There was a significant direct negative relationship between the FMPS Organisation dimension and both main NSSI functions. This study draws attention to an increasing trend and the extremely high NSSI prevalence rate among community adolescents. Adolescents with perfectionistic concerns are at heightened risk for anxiety disorders, which can increase their vulnerability to NSSI engagement.

## 1. Introduction

Nonsuicidal self-injury (NSSI), which refers to deliberate self-inflicted harm without suicidal intent [1,2,3], has become a serious public health concern among adolescents. Although NSSI is a prevalent phenomenon, it remains hidden in many cases [4], and stigmatisation of it is common among adolescents [5]. The lifetime prevalence of NSSI among adolescents ranges from 17.1% to 46.5% in community samples [6,7,8,9,10,11] and from 51.3% to 82.4% in clinical adolescent populations [12,13,14]. Moreover, the prevalence of NSSI in adolescents has been on the rise over the past 15 years [4,15].

NSSI engagement serves several psychological functions, and most individuals use multiple functions [16,17,18,19]. Studies focusing on the psychological functions of NSSI support two main factors related to NSSI functionality: (1) intrapersonal (e.g., to manage one’s uncomfortable internal state) and (2) interpersonal (e.g., to influence one’s social environment) motivation [20], and suggest that intrapersonal motives are more prevalent and strongly associated with internalising and externalising mental symptoms than are interpersonal motives [17,19,20,21]. 

Perfectionism is a potential risk factor for engaging in NSSI [22]. It is conceptualised as a multidimensional construct (with intra- and interpersonal aspects) [23,24], and commonly defined as “setting excessively high performance standards, accompanied by overly critical self-evaluations” [23] (p. 450). Factor analytic studies have distinguished two main factors: (1) maladaptive evaluation concerns (maladaptive) and (2) positive achievement striving (adaptive) [25,26,27,28,29]. In recent years, the prevalence rate of maladaptive perfectionism among community adolescents has also reached alarming levels: 22% to −38% [28,29,30,31,32], and, similar to NSSI, perfectionism also is showing an upward tendency [4,15,33,34].

Until now, only a few studies have explored the function of NSSI among perfectionistic adolescents; moreover, some results are inconsistent. Nock and Prinstein (2005) emphasised the role of the social (interpersonal) function of self-injury and found that psychiatric adolescents who perceive unrealistic high expectations from their environment tend to use self-injury to get support from others or to avoid those perceived expectations [35]. Meanwhile, inconsistent with that, Claes et al. (2012) found that among women diagnosed with eating disorders (EDs), patients who perceived parental criticism had a negative relationship with the cry-for-help function of NSSI behaviour [36]. Other findings, with a community adolescent sample [37] and women ED patients [36], have supported the role of the intrapersonal motivation of NSSI related to self-critical perfectionistic concerns. This evidence suggests that unhealthy perfectionistic people tend to use NSSI in order to handle strong negative emotions. Results among perfectionistic people related to NSSI function may be influenced by the age of the study population and by the mental disorders examined [22].

Related to the possible pathway between the two phenomena, few studies have focused on the examination of direct and indirect mechanisms between NSSI and perfectionism, and several aspects remain unclear [22]. Some results indicate a direct relationship between NSSI and perfectionism [36,38,39] and an indirect effect through rumination and negative affect [39]. Gu et al. [8], in a study of community adolescents, found that psychological distress has a mediating effect on the association between the two phenomena. This suggests that there is an indirect relationship between maladaptive perfectionism and NSSI, which is mediated by emotional distress symptoms [8]. It may also imply that NSSI is used as a maladaptive coping strategy to reduce or communicate unwanted emotional states.

Regardless of the fact that the positive perfectionism dimension is characterised by more adaptive outcomes [27], several studies and metanalytic results have indicated that both perfectionism dimensions (adaptive and maladaptive) [32,40,41,42,43,44,45,46,47,48] and NSSI [12,49,50] have a significant relationship with several internalising and externalising mental disorders. In addition, both phenomena mean risk factors related to suicidal behaviour [6,12,44,51,52,53,54,55]. Maladaptive perfectionism (evaluation concerns) plays an important role in NSSI engagement [22]. Individuals with maladaptive perfectionism and NSSI tend to be highly self-critical [23,56,57,58], and unhealthy perfectionistic adolescents and individuals with NSSI engagement report similar difficulties in emotion regulation [59,60]. People with maladaptive perfectionism tend to react to failure with elevated levels of shame, guilt, depression, anxiety, and anger [61,62,63], and these strong negative emotions [61,62,63] may motivate them to self-harm [64]. These results confirm findings that emphasise that perfectionistic individuals tend to engage in NSSI because of self-punishment, self-torture, and cry-for-help motives [36], and affect regulation and the self-punishment function of NSSI are the most common motivations for this behaviour [2,19], which play an important role in shame coping [65,66].

Adolescents with a history of NSSI rate their family life satisfaction, physical and mental health, and global well-being significantly lower than adolescents without NSSI [12]. Given the high prevalence of NSSI and due to its significant association with a range of several comorbid internalising and externalising mental disorders [12,49], especially with suicidal behaviour [6,67], NSSI has been recognised as a long-lasting public health problem among adolescents [49,68]. It is critical to explore this behaviour to develop interventions and treatments in order to support those struggling with NSSI engagement [19,69]. 

Although both NSSI and perfectionism are public health concerns and have a high prevalence rate in the adolescent years, more detailed explorations of NSSI behaviour among community adolescent samples in connection with perfectionism are lacking. Much of the literature has focused on adults, although prevalence rates related to both phenomena are high in the adolescent years. On the basis of the previous literature, little evidence related to perfectionism and NSSI, especially among community adolescent samples, is available, which raises further issues. The primary aim of this study was to address gaps in the literature by exploring the relationship between perfectionism dimensions and different NSSI functions and examining the potential mediating effects of different mental disorders on this relationship. Our hypotheses were as follows:

**Hypothesis** **1.** The association between perfectionism dimensions and NSSI is mediated by comorbid mental disorders.

**Hypothesis** **2.** Maladaptive perfectionism is more strongly associated with the intrapersonal function of NSSI than interpersonal motivation, and this relationship is mediated by higher levels of mental disorders.

## 2. Materials and Methods

### 2.1. Ethics

This study was conducted in accordance with the Declaration of Helsinki and approved by the National Scientific and Ethical Committee of the Medical Research Council, Hungary (Protocol No. 54023-5/2018/EKU, IV/8167-3/2020/EKU). This study was approved on 20 November 2018 (Protocol No. 54023-5/2018/EKU). After receiving ethical approval, data collection started on 5 June 2019.

Both the adolescents and their parents gave their written informed consent after the nature of this study had been explained. Participants were assured that all collected data were treated with strict confidentiality. In case of perception of an acute suicide risk based on a structured psychiatric diagnostic interview, parents and adolescents were informed and referred to the health care system. 

### 2.2. Participants and Data Collection

The inclusion criteria were being aged between 13 and 18 years. The exclusion criteria were conditions preventing the completion of self-reported questionnaires (intellectual disability or serious mental states, e.g., delirium). 

Our study has a cross-sectional study design. Sample was conducted with a Hungarian convenience sample of adolescents. Our research group developed a new school mental health preserving prevention program [70]. Several schools contacted us to request this program, as teachers perceived that students might have mental difficulties. All participants took part in our study before their participation in the prevention program. Participants were recruited from Hungarian secondary schools during a recruitment period spanning 5 June 2019 to 23 September 2022. The instruments consisted of a structured diagnostic interview (see below) and self-report questionnaires. After informed consent was obtained, participants were assessed with the structured diagnostic interview by a trained researcher in separate classrooms in the school. The digital version of the self-report questionnaires was completed in the computer rooms of the schools in the presence of research staff, providing the opportunity to ask questions. Parents/caregivers received the link to the online parent self-report questionnaire by email. When technical possibilities were not available in the schools, questionnaires were carried out on paper. Following the outbreak of the COVID-19 pandemic (March 2020), it was forbidden to go personally to schools due to the safety regulations, which made in-person data collection impossible. We had to modify our data collection process and completely switch to online data collection. Our study was approved again regarding the methodological changes by the National Scientific and Ethical Committee of the Medical Research Council, Hungary (IV/8167-3/2020/EKU). After gaining the new, extended ethical approval, adolescents in the schools were informed through an introduction video of our research group, and informed consent was shared and gained with help of teachers and principals of the schools. After the adolescents and their parents/caregivers gave their written informed consent, we contacted them by phone, and they were also informed verbally about how online data collection would be conducted. In the case of the structured diagnostic interview, a member of our research staff sent a link to every adolescent individually via email, and the structured diagnostic interview was completed in a two-person situation with help of online video connection at a previously scheduled appointment. Regarding the self-report questionnaires, a link to the digital version of the self-report questionnaire package was sent individually by email to the adolescents. The self-report questionnaires were then completed in the computer rooms of the schools. During the time of the completion, our research staff ensured online video connection and provided opportunity for the participants to ask questions if necessary. Twenty-four adolescents were tested online by structured diagnostic interviews. After we had the opportunity to visit the schools in person again, we continued our data collection personally. When the COVID-19 pandemic regulations made it possible to visit the schools in person again, we continued our data collection personally.

A total of one hundred eighty-three 13- to 18-year-old participants gave written informed consent; of these, five adolescents withdrew their participation, and thirteen were not available for data collection despite their prior consent (e.g., they were absent from school on the days of the data collection). During the COVID-19 pandemic, we collected our data online, and even though we had arranged a scheduled appointment for the interview with the adolescents, it happened that they were not available at the time, and we were unable to reach them again later. Overall, compared to the in-person data collection, there were more dropouts during the online data collection, making it more difficult to reach participants in this way. Another six were excluded because three of them were older than eighteen; three other individuals took part twice in this study, and thirteen completed only half of the questionnaire package. Overall, one hundred forty-six adolescents completed the questionnaires. The response rate of parents/caregivers was 44.24% (seventy-three individuals).

### 2.3. Measures

Age and gender were assessed with the adolescents’ self-reported questionnaires. Mental disorders, according to criteria in the *Diagnostic and Statistical Manual of Mental Disorders, Fifth Edition* (DSM-5) [1], were evaluated with the modified version of the Hungarian Mini International Neuropsychiatric Interview Kid 7.0.2 (MINI–KID) [71,72,73,74,75,76], which assess the major child and adolescent psychiatric disorders and suicidal behaviour. Although suicidality is not an official diagnosis, the DSM-5 [1] included Suicidal Behaviour Disorder (SBD) as a “condition for further study” because psychiatrists and researchers had recognized the importance of suicidal behaviour as a psychiatric condition. This proposal might lead to SBD being included in a later edition. We assessed the suicidal behaviour (suicidal thoughts, plans, attempts) with the MINI–KID. MINI–KID assesses suicidal behaviour very sensitively with several questions related to suicidal thoughts, ideation, plans, attempts. According to the MINI–KID, if a participant answered YES to any of the questions regarding suicidal thoughts, plans, attempts, the case was classified as suicidal behaviour. The prevalence rates of suicidal behaviour in the current study thus indicate how many adolescents answered YES to any of these questions on thoughts, ideations, plans, attempts on the suicidal behaviour spectrum. The phrase suicidality is used as a synonym for suicidal behaviour within the Results section. Each participant received a code number at the start of this study. This code–decode system was used to identify participants if the answers in the MINI–KID structured diagnostic interview indicated the possible presence of an acute suicidal risk. In this case, the participant was immediately contacted by the child- and adolescent psychiatrist member of our team, and a clinical interview was conducted on-site to exclude or confirm the presence of an acute suicidal risk. If the acute risk was confirmed, the child- and adolescent psychiatrist specialist from the local health care system contacted the parent/caregiver of the participant by phone to give detailed information about emergency care, and the participant was referred to the specialised health care system.

MINI–KID is a comprehensive structured diagnostic interview, which was administered by trained and continuously supervised interviewers. The interrater and test–retest reliability of the Hungarian version of the MINI–KID [72] were adequate. Criterion validity was acceptable, and the sensitivity and specificity in the majority of examined disorders were reported as very good or good [72]. In our study, MINI–KID symptoms were assessed with Kruder–Richardson formula (KR-20). In general, a score of above 0.5 signals an acceptable level of reliability [77]. In our study, KR-20 related to mood disorders: 0.45, anxiety disorders: 0.52, substance use disorders: 0.55, attention-disruptive disorders: 0.51, Tic: −0.10, eating disorders: 0.18.

NSSI was assessed with the Hungarian version of the Inventory of Statements about Self-Injury (ISAS–HU) Part I and II [17,78]. The first section of the ISAS–HU [17,78] measures the lifetime frequency of 12 different NSSI methods performed intentionally without suicidal intent, including cutting, carving, burning, wound picking, banging or hitting self, biting, needle-sticking, pinching, rubbing skin against rough surfaces, hair pulling, severe scratching, and swallowing chemicals. Further questions explore descriptive features regarding NSSI: the age of first NSSI act and date of the last episode, the experience of physical pain during NSSI, whether self-injury behaviour was conducted alone or around others, time between the urge and the act of NSSI, and whether the respondent wanted to stop the self-injury. The second section assesses 13 different functions of self-injury behaviour (e.g., affect regulation, anti-dissociation, anti-suicide, autonomy, interpersonal boundaries, interpersonal influence, marking distress, peer bonding, self-care, self-punishment, revenge, sensation seeking, and toughness) on a 3-point scale ranging from 0 (not relevant) to 2 (very relevant). The 13 different functions were divided into two superordinate factors: (1) intrapersonal function and (2) interpersonal function [17,20,79]. The ISAS–HU has good reliability coefficients for both intrapersonal (Cronbach’s α = 0.84) and interpersonal (Cronbach’s α = 0.82) functions [78]. 

Perfectionism was assessed with the Frost Multidimensional Perfectionism Scale (FMPS) [23], one of the most frequently used questionnaires for measuring perfectionism [80]. It evaluates the adaptive and maladaptive dimensions of perfectionism with 35 items that are rated on a 5-point Likert scale that ranges from 1 (strongly disagree) to 5 (strongly agree). Six scales were designed to assess measuring perfectionism: Concern Over Mistakes (CM; negative critical reactions to failure), Doubts About Actions (DA; doubting one’s ability to accomplish things), Parental Expectations (PE; the perception that one’s parents expect extremely high performance), Parental Criticism (PC; the belief that one’s parents are extremely critical of one’s ability/performance), Personal Standards (PS; the setting of high standards of performance and goals), and Organisation (O; the importance of order and neatness). The internal consistency (Cronbach’s α) of the six scales ranged from 0.77 to 0.93, and the reliability (Cronbach’s α) of the total FMPS scales was 0.90 [23]. 

### 2.4. Statistical Analysis

Statistical analysis was performed using R (Version 3.5.1; R Foundation for Statistical Computing, Vienna, Austria). The following grouped diagnoses were involved in our analysis: (1) suicidality; (2) mood disorders: major depressive episode, hypo/manic episode; (3) anxiety (and related) disorders: panic disorder, agoraphobia, separation anxiety disorder, social anxiety disorder, specific phobias, generalised anxiety disorder, posttraumatic stress disorder, and obsessive–compulsive disorder; (4) attention-disruptive disorders: attention-deficit/hyperactivity disorder, conduct disorder, oppositional defiant disorders; (5) substance use disorders: alcohol use disorder, substance use disorder; (6) tic; (7) psychotic disorders; (8) EDs (anorexia nervosa, bulimia nervosa); (9) autism spectrum disorder (ASD); and (10) borderline personality disorder (BPD). We categorised the variables related to mental disorders in a binary fashion; they were coded 1 in the presence of a specific diagnosis and 0 otherwise. 

In our analysis, NSSI was also assessed with the presence or absence of any ISAS–HU self-injury methods (dichotomous variable). Regarding the intrapersonal and interpersonal motivation categories, we used the ISAS–HU categories [78] in which, contrary to the original model, the anti-suicide function belongs to interpersonal function [17]. According to the ISAS–HU [78], interpersonal motivations refer to anti-suicide, interpersonal boundaries, self-care, sensation seeking, peer bonding, interpersonal influence, toughness, autonomy, and revenge, and the intrapersonal function refers to affect regulation, self-punishment, anti-dissociation, and marking distress. 

Related to the two main identified structures of perfectionism [25,26,27,28,80,81,82], the CM, DA, PE, and PC subscales from the FMPS have been considered to encompass maladaptive evaluation concerns (i.e., unhealthy perfectionism) and the PS and O subscales to encompass healthy perfectionism, positive striving [25,27]. A further factor analytic investigation related to the FMPS subscales suggested that in addition to the Concern Over Mistakes and Doubts dimension (which comprises the CMD, CM, and DA subscales), the parental subscales (PE and PC) may create a separate parental dimension (Parental Expectations and Criticism [PEC]) [29], which may distinguish the developmental factor of perfectionism [82]. Investigations related to the PS subscales suggest that personal standards may be part of both the healthy and unhealthy perfectionism constructs [29,44,82,83]. Some evidence suggests that the O subscale may be a separate factor from two core perfectionism facets [28,80], whereas others have reported a positive correlation between the O and PS scales. The O subscale has been observed to be a positive characteristic of perfectionism, and a high score on it may separate positive from negative perfectionistic people [28,81,84]. Following previous studies [28,84,85,86], we used four dimensions to explore the nature of perfectionism: (1) CMD (FMPS–CM and DA subscales), (2) PEC (FMPS–PE and PC subscales), (3) the FMPS–PS subscale, and (4) the FMPS–O subscale. Variables connected to perfectionism and NSSI intrapersonal and interpersonal motivation were count variables.

We now discuss descriptive statistics. In testing both hypotheses, an α level below 0.05 was considered to be significant. The relationships among the perfectionism dimensions, mental disorders, and NSSI were examined with multiple logistic regression models. We used a negative binomial regression analysis to analyse the relationship between maladaptive perfectionism and NSSI intrapersonal and interpersonal motivation and mental disorders. We used Baron and Kenny’s three-step method [87] to show potential mediation effects. We assessed the joint effects of the FMPS variables with a Wald test. Mixed graphical network models regularised based on the Extended Bayesian Information Criterion were applied to further explore the relationships among the variables, namely, whether the different mental disorders mediated the association between the perfectionism dimensions and NSSI and NSSI functions. Network modelling provides a test of the potential explanatory mechanism between examined variables. It enabled us to show the different pathways between examined variables, how mental disorders, perfectionism dimensions, and NSSI can influence each other in different ways. Network modelling is important in clinical study providing a comprehensive model related to complex interrelationships [88,89]. For network estimations, the bootnet package was used [88]. In this model, we used the dichotomous variables of NSSI intrapersonal and interpersonal functions. Information related to the literature background of network modelling was detailed in a previous study [12].

## 3. Results

### 3.1. Sample and Descriptive Statistics

The final study sample consisted of 146 adolescents (28.77% males [*n* = 42], 71.23% females [*n* = 104]). The mean age was 15.76 years (SD = 1.16). In the study group, 90 adolescents (61.64%) engaged in NSSI, and 71.11% of them were girls. The prevalence rates of NSSI were 61.54% among girls and 61.90% among boys. There was no significant gender difference related to the prevalence of NSSI, χ2 (1, *N* = 134) = 0.00, *p* > 0.05 (*p* = 0.99). Table 1 shows the prevalence rates of different NSSI methods by gender. There are no significant gender differences related to NSSI methods. Table 2 shows the prevalence rates of mental disorders among the community sample of adolescents and the prevalence rates of mental disorders among adolescents who engaged in NSSI.

A total of 12 of the 146 participants who reported a history of NSSI did not complete the second section of the ISAS–HU related to NSSI function; therefore, we analysed Hypothesis 2 in a sample of 134 adolescents (girls: *n* = 96; 71.64%). The mean age was 15.78 years (SD = 1.18). In this study group, 68 adolescents (50.74%) engaged in NSSI. Fifty-five (80.88%) reported using both NSSI interpersonal and intrapersonal functions, and thirteen (19.12%) used the intrapersonal or interpersonal function. Overall, 88.23% (*n* = 60) adolescents reported using the intrapersonal motivation of NSSI, and 92.64% (*n* = 63) reported using the interpersonal motivation. There was no significant difference related to the prevalence of NSSI functions (intrapersonal, interpersonal), χ2 (1, *N* = 134) = 83.84, *p* > 0.05 (*p* = 2.20). 

### 3.2. Statistical Analysis Related to Our Hypotheses

Related to our hypotheses, we now discuss the most relevant significant associations among the examined variables.

#### 3.2.1. Regression Analysis

Table 3 presents the results of Step 1 of the multiple regression analyses and the negative binomial regression analysis.

The joint effect of the FMPS dimensions was marginally significant in predicting NSSI, χ2(4) = 9.15, *p* = 0.06. The FMPS–O dimension significantly and negatively predicted NSSI, B = −0.11, t(129) = −2.07, *p* = 0.04, and the FMPS–CMD dimension positively and marginally predicted NSSI, B = 0.04, t(129) = 1.70, *p* = 0.09. 

According to the negative binomial regression analysis, the FMPS–CMD dimension significantly and positively predicted both NSSI intrapersonal motivation, B = 0.05, t(117) = 2.94, *p* < 0.01 (*p* = 0.0038), and NSSI interpersonal motivation, B = 0.04, t(117) = 2.58, *p* < 0.01 (*p* = 0.011), with comparable effect sizes. Of the maladaptive dimensions, the FMPS–O dimension significantly and negatively predicted NSSI interpersonal motivation, B = −0.09, t(117) = −2.82, *p* < 0.01 (*p* = 0.006; Table 3).

Table 4 presents the results of Step 2 of the multiple regression analyses and the negative binomial regression analysis.

The joint effect of mental disorders was marginally significant in predicting NSSI engagement, χ2(10) = 17.16, *p* = 0.07. The association between the FMPS–O dimension and NSSI engagement was significant even after controlling for the effect of mental disorders, B = –0.14, t(119) = –2.36, *p* = 0.02. The association between the FMPS–CMD dimension and NSSI engagement became completely insignificant after controlling for the effect of mental disorders. According to our results, only anxiety disorders predicted NSSI engagement significantly and positively, B = 2.39, t(119) = 3.09, *p* < 0.01 (*p* = 0.0025). 

The results of a negative binomial regression analysis related to Hypothesis 2 indicated that the association between NSSI intrapersonal and interpersonal motivation and the FMPS–CMD dimension became insignificant after controlling for the effect of mental disorders. Only anxiety disorders predicted significantly and positively both NSSI intrapersonal motivation, B = 1.03, t(107) = 2.79, *p* < 0.01 (*p* = 0.006), and NSSI interpersonal motivation, B = 1.13, t(107) = 3.21, *p* < 0.01 (*p* = 0.001), with comparable effect sizes. Mood disorders, EDs, and BPDs marginally and positively predicted NSSI intrapersonal motivation. The relationship between the FMPS–O dimension and NSSI intrapersonal motivation, B = −0.08, t(107) = −2.13, *p* < 0.05, and NSSI interpersonal motivation, B = −0.09, t(107) = −2.75, *p* < 0.05 (*p* = 0.006), showed a significant negative association even after controlling for the effect of mental disorders (Table 4).

Table 5 presents the results of Step 3 regarding the regression analysis. The FMPS dimensions jointly predicted anxiety disorders, χ2(4) = 19.01, *p* < 0.001. 

The FMPS–CMD dimension significantly and positively predicted anxiety disorders, B = 0.09, t(129) = 3.07, *p* < 0.01 (*p* = 0.002). The FMPS–PEC dimension marginally and positively predicted anxiety disorders, B = −0.06, t(129) = 1.83, *p* = 0.07. The FMPS–O dimension had no significant association with anxiety disorders (Table 5).

The strongest significant positive association can be found between the FMPS–PS and FMPS–CMD dimensions (ρs = 0.68, *p* < 0.001) and between the FMPS–CMD and FMPS–PEC dimensions (ρs = 0.49, *p* < 0.001; Table 6).

#### 3.2.2. Network Analysis

After the regression analysis, we explored the complex relationship among NSSI, perfectionism dimensions, and mental disorders with a regularised psychological network model. Unfortunately, because of the small sample size, the effects in the network model were not significant, but the findings revealed directions and patterns similar to those in the regression analysis.

**Figure 1:**
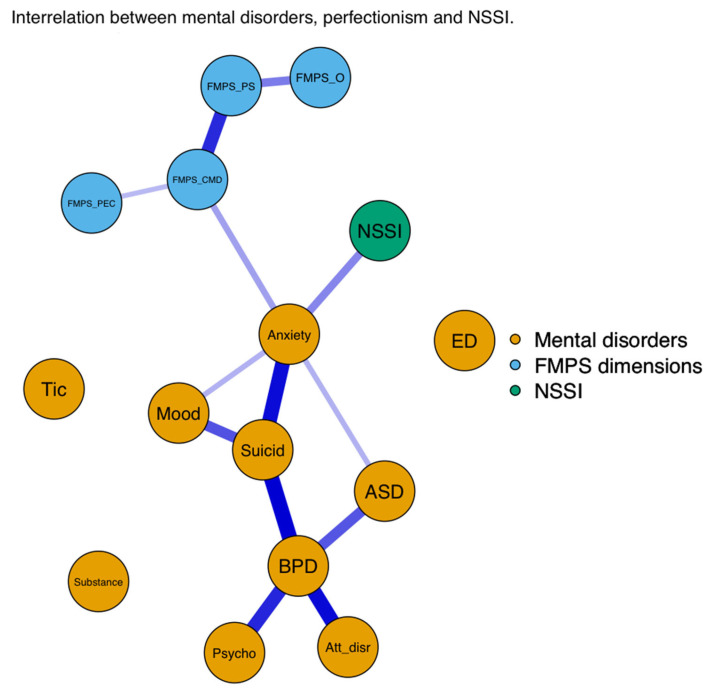
Associations among NSSI, FMPS dimensions, and mental disorders.

Note: γ (gamma) = 0.125. Anxiety = anxiety disorders; ASD = autism spectrum disorder; Att_disr = attention-disruptive disorders; BPD = borderline personality disorders; CMD = Concern Over Mistakes and Doubts About Actions subscales; ED = eating disorders; FMPS = Frost Multidimensional Perfectionism Scale; Mood = mood disorders; NSSI = nonsuicidal self-injury; O = Organisation subscale; PEC = Parental Expectations and Criticism subscales; PS = Personal Standards subscale; Psycho = psychotic disorders; Substance = substance use disorders; Suicid = suicidality.

**Figure 2:**
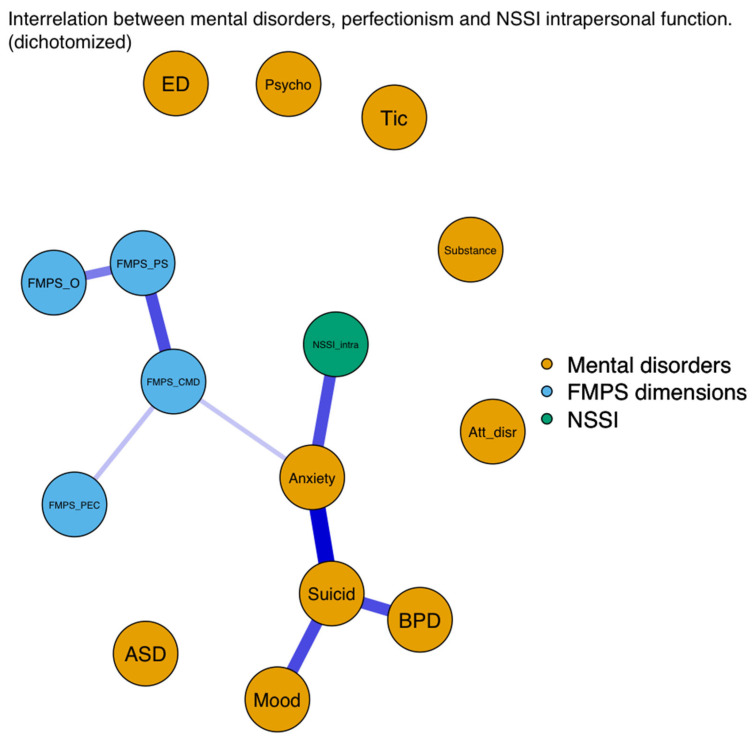
Associations among NSSI intrapersonal function, FMPS dimensions, and mental disorders.

Note: γ (gamma) = 0.125. Anxiety = anxiety disorders; ASD = autism spectrum disorder; Att_disr = attention-disruptive disorders; BPD = borderline personality disorders; CMD = Concern Over Mistakes and Doubts About Actions subscales; ED = eating disorders; FMPS = Frost Multidimensional Perfectionism Scale; Mood = mood disorders; NSSI = nonsuicidal self-injury; NSSI_intra = intrapersonal motivation of nonsuicidal self-injury; O = Organisation subscale; PEC = Parental Expectations and Criticism subscales; PS = Personal Standards subscale; Psycho = psychotic disorders; Substance = substance use disorders; Suicid = suicidality.

**Figure 3:**
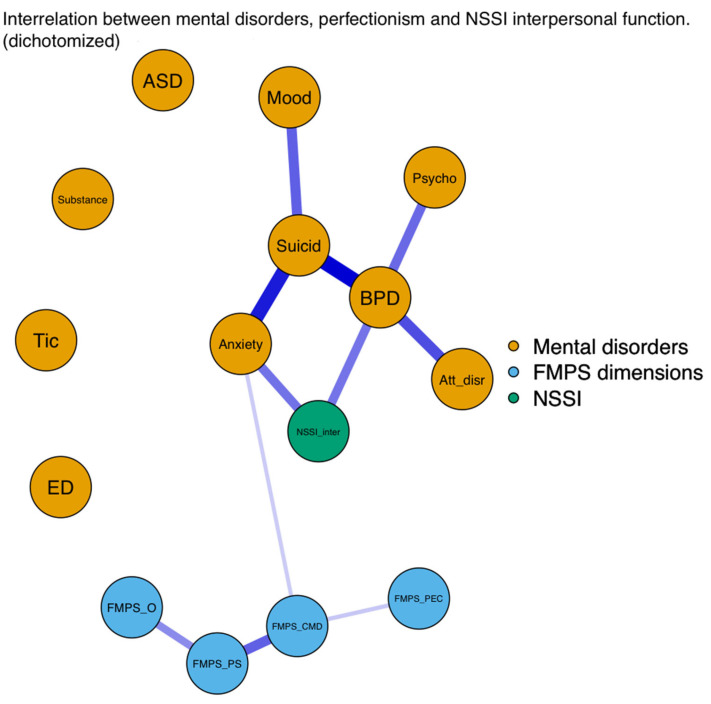
Associations among NSSI interpersonal function, FMPS dimensions, and mental disorders.

Note: γ (gamma) = 0.125. Anxiety = anxiety disorders; ASD = autism spectrum disorder; Att_disr = attention-disruptive disorders; BPD = borderline personality disorders; CMD = Concern Over Mistakes and Doubts About Actions subscales; ED = eating disorders; FMPS = Frost Multidimensional Perfectionism Scale; Mood = mood disorders; NSSI = nonsuicidal self-injury; NSSI_inter = interpersonal motivation of nonsuicidal self-injury; O = Organisation subscale; PEC = Parental Expectations and Criticism subscales; PS = Personal Standards subscale; Psycho = psychotic disorders; Substance = substance use disorders; Suicid = suicidality.

With regard to the statistical results, we focused only on the significant association between the examined variables; therefore, we provide Figure 1, Figure 2 and Figure 3 related to the results of the network analysis, and detailed information related to the numeric data of the network model can be found in the Appendix A (Table A1, Table A2 and Table A3).

In sum, the results of the network analysis showed that of the examined mental disorders, anxiety disorders had a direct association with NSSI engagement, and NSSI intrapersonal function also had a direct association with BPDs. Of the four perfectionism dimensions, only the FMPS–CMD dimension had a direct association with anxiety disorders that lead to NSSI. In addition to NSSI, anxiety disorders had a strong positive association with suicidal behaviour. With regard to the perfectionism dimensions, the strongest positive association was found between the FMPS–PS and FMPS–CMD dimensions. 

## 4. Discussion

Many lines of evidence suggest that, among the community adolescent population, NSSI has reached an extremely high prevalence rate of 17.1% to 46.5% [6,7,8,9,10,11,21], and findings from longitudinal cohort studies and recent review studies show an upward trend [15,90]. Meta-analytic results related to the past decade show an increasing trend toward more serious self-injuries among nonclinical adolescents [4]. Related to this phenomenon, Xiao et al. (2022) mentioned the important role of the development of social networking sites, growing learning expectations for youth, and maladaptive coping mechanisms, in addition to changes and problems in personal relationships. Social networking sites (e.g., Instagram) have an important priority in most adolescents’ daily lives [91,92], and the number of online sites that promote self-injury activities (e.g., NSSI wounds photos, videos, posts) is growing and provides the opportunity for youth to contact other people who engage in NSSI [91,92]. Social positive reinforcement may be an important factor in maintaining the posting of NSSI content online. Severe wound pictures might lead to elevated levels of interest and empathetic comments, which can affect bidirectional and encourage further posting of severe self-injury [92]. Vulnerable adolescents tend to use social networking websites to benefit from social support [90], and other self-injurers online friends may encourage them to self-harm [92,93]. These virtual self-injuring communities can serve as a potential identity-formation outlet for those who have problems with this developmental process [92,94], and identity confusion is a significant predictor of NSSI engagement among adolescents [38]. Although social media activities have many negative consequences (negative comments, encouragement to self-harm, triggering, competition), there are also benefits for people who self-injure (positive sense of community, reduction of social isolation, anonymity, support, reduction of self-injury urges) [95,96].

Our results may support this increasing trend in community adolescents; for example, in our nonclinical sample, 61.64% of adolescents reported engaging in NSSI. Both girls (61.54%) and boys (61.90%) reported extremely high and approximately equal prevalence rates of NSSI, even though previous meta-analytic results have emphasised higher NSSI engagement among girls than boys [4,97]. On the basis of some additional analysis related to the prevalence rate of NSSI, and the fact that this high prevalence rate cannot be explained by a COVID-19 pandemic effect during our recruitment period, the prevalence rate in our adolescent sample before the first COVID-19 wave was 68.09%, and during and after the second and third waves, it was 43.33%—a 43.33% prevalence rate similar to the recent rates to another Hungarian community sample (41.2%) [21] and to other international results in Swedish, 41.6% [8], Chinese, 47.1% [98], and Brazilian, 45.3% [99], community adolescent samples. The 68.09% NSSI prevalence rate before the first COVID-19 wave in our sample is higher than the recent Hungarian and international prevalence rate, and the exact reason for this high prevalence rate is unknown. We can provide only a hypothetical explanation. Our research group works in the field of school-based adolescent mental health improvement and suicide prevention [100,101]. We developed a new school-based mental health-promoting prevention program [70], and several schools contacted us to request this prevention program, as the teachers perceived that students might have mental problems. All participants took part in our study before the prevention program. The high prevalence rates of NSSI and mental disorders derived from this population and our findings draw attention to how much mental health-preserving prevention programs are needed at schools. Our results represent the mental state of those high school students where the sensitive attention of teachers recognised the potential problems.

Many studies have indicated that adolescence is a sensitive developmental period that drives both neural and social changes and increases vulnerability to emotion regulation problems and psychiatric disorders (e.g., depression and anxiety) [102,103]. Similar to previous studies [12,49,50,51] our results also emphasise the high prevalence rate of internalising and externalising psychiatric disorders among adolescents who engage in NSSI. An important highlighting result is that the highest comorbid mental disorder in the NSSI group is ASD (52.22%); however, questions on the MINI–KID about ASD diagnoses mainly serve to exclude the diagnosis rather than establish it, so further investigations in this field are needed [71,72,73,74,75,76]. Our findings are in line with those of previous studies [104,105] and suggest that self-injurious adolescents tend to report higher levels of depressive symptoms, anxiety, and suicidality. In our community adolescent sample, 38.89% of youth who engaged in NSSI also had anxiety disorders, and 36.67% reported mood disorders and suicidality. 

To our knowledge, this study is the first to examine all relevant mental disorders among adolescents in connection with different perfectionism dimensions and NSSI engagement. Investigating the complex nature of the association between NSSI and perfectionism provides relevant information for prevention and intervention regarding NSSI engagement. Hypothesis 1 stated that the association between perfectionism dimensions and NSSI is mediated by comorbid mental disorders. Our results offer preliminary evidence supporting the mediating effect of mental disorders, especially anxiety disorders, on the relationship between maladaptive perfectionism and NSSI engagement; therefore, our Hypothesis 1 was supported, but additional research is necessary to confirm these results with larger samples. The importance and novelty of the topic are demonstrated by the fact that only a few recent studies have explored the potential moderating and mediating effect between the two phenomena, and our findings suggest that individuals with perfectionistic concerns and doubts are at a greater risk for anxiety disorders and therefore a greater risk for NSSI. These findings confirm previous evidence that has emphasised the mediating role of negative affect, psychological distress between the two phenomena [8,39], and that maladaptive perfectionism is one of the main key factors related to anxiety symptoms [106,107]. 

Although previous studies have suggested that the intrapersonal functions of NSSI engagement are more prevalent, and more strongly associated with internalising and externalising mental symptoms, than interpersonal motives [17,19,20,21], the adolescents in our sample used both NSSI motivations at approximately equal rates. In addition, similar to previous studies [16,17,18,19], the majority (80.88%) of them used multiple functions regarding NSSI engagement. In contrast to Hypothesis 2—maladaptive perfectionism is more strongly associated with the intrapersonal function of NSSI than the interpersonal motivation, and this relationship is mediated by higher levels of mental disorders—our results did not support Hypothesis 2 and showed that unhealthy perfectionistic adolescents tend to commit self-harm for both intrapersonal and interpersonal motivations to a similar extent and that anxiety disorders have a central role in this mechanism. Although previous results among community adolescents have shown that maladaptive perfectionistic adolescents tend to use self-injury primarily because of intrapersonal motivation [37], our results demonstrate that the relationship between maladaptive perfectionistic tendencies and NSSI engagement seems to be independent of self-injury motives and that unhealthy perfectionistic adolescents tend to use self-injury to the same extent to escape from negative emotional states and as a means of communicating or exerting interpersonal influence. We should mention that there is a methodological difference between the present study and Reinhardt et al.’s because Reinhardt et al. (2021) used the original categorisation of NSSI motives regarding the ISAS [21] in which anti-suicide motives belonged to intrapersonal functionality. After the Hungarian adaptation of the ISAS [21] was published, we assessed the NSSI function according to the ISAS–HU, in which the anti-suicide function belongs to interpersonal motivation. The anti-suicide function of NSSI serves as a coping mechanism against suicidal thoughts and attempts [2,19]. More than one-third of our community adolescents with a history of NSSI reported suicidal behaviour according to a structured psychiatric interview, so the anti-suicide function may have influenced the results between the two Hungarian studies. We confirmed it with additional analysis. We categorised again the NSSI function according to the original categorisation of NSSI motives regarding the ISAS [17]. Our additional results confirmed previous studies, which emphasised the higher rate of NSSI intrapersonal function [17,19,20,21] and the stronger association between NSSI intrapersonal function and maladaptive perfectionism [36,37] (see Table 5 and Table A4 and Table A5 in Appendix A). Overall, 95.58% (*n* = 65) of adolescents who engaged in NSSI (*n* = 68) reported using the intrapersonal motivation of NSSI, and 85.29% (*n* = 58) reported using the interpersonal motivation. There was a significant difference related to the prevalence of NSSI functions (intrapersonal, interpersonal), χ2 (1, *N* = 134) = 77.69, *p* < 0.0001. According to this original categorisation of NSSI motives regarding the ISAS [17], our additional findings supported Hypothesis 2.

The strong association between anxiety disorders and NSSI engagement has been proven with meta-analytic review evidence [108], and one of the main functions of NSSI is emotion regulation and the reduction in anxiety [19,109]. Anxiety disorders among adolescents are a relevant predictor of experiential avoidance [110], and the Experiential Avoidance Model (EAM) [111] emphasises that NSSI behaviour serves as a means to escape undesirable emotional experiences, and the temporary relief after self-harm repeatedly reinforces this maladaptive behaviour. It is essential that individuals with anxiety symptoms do not negatively judge their internal emotional states because this attitude can decrease the risk related to NSSI engagement [112]; however, the repetitive self-critical thinking and rumination of maladaptive perfectionistic individuals increase psychological distress and negative emotions [39,56]. The Emotional Cascade Model (ECM) [113] emphasises the mutually reinforcing mechanism between ruminative thoughts and negative emotions. NSSI breaks this aversive reciprocal cycle and distracts one’s focus away from negative emotional states with physical acts of self-injury [113]. Our results confirm previous evidence that has emphasised that maladaptive perfectionism may play a significant role in these emotional cascades [39].

Maladaptive perfectionistic adolescents try to seem perfect and competent in every daily situation in school, but their perfectionist pursuits often result in rejection and bullying from peers [45], as well as social isolation, and the social hopelessness of these adolescents can elevate the risk for several mental disorders (e.g., anxiety, depression) [45] and suicidal risk [114]. Maladaptive perfectionistic people tend to be seen as invulnerable and try to hide their real emotions after a failure and, because of a high level of distress, try to escape from situations in which they have to speak in front of classmates [115,116]. The perfectionistic self-presentation of adolescents, the need to look perfect and invulnerable to other people, is a significant risk factor related to anxiety symptoms [115]. Social network sites also provide an opportunity for superficial contact without really showing oneself [116,117]. Unhealthy perfectionist adolescents make great efforts to keep any sign of their anxiety invisible, and they tend to avoid seeking help; therefore, it is really hard to recognise the urgent need for help when they are hiding behind a mask of perfection [116].

Our findings confirm previous results that have suggested that the importance of order and neatness may be a healthy dimension of perfectionism [29,84] and a protective factor against NSSI engagement [102] independent of any mediating effect of mental disorders. This may mean that the importance of order and neatness refers to the ability to manage and control one’s daily life and emotional experiences, and adolescents with low Organisation subscale scores may perceive their feelings and everyday situations as unmanageable, and thus NSSI behaviour gives them a “sense of control” [104] (p. 583). 

In summary, our results confirm and indicate that adolescents who report a higher rate of maladaptive perfectionism concerns are more likely to engage in NSSI, which is consistent with previous systematic review evidence that has highlighted the important role of perfectionistic concern related to NSSI engagement [22]. Our study serves implications for prevention and intervention related to adolescent NSSI. Prevention and intervention should focus on the reduction of potential risk factors related to NSSI. Our findings emphasise that teachers and professionals should pay attention to unrealistic high standards that parallel with actual ability and should support reachable goal setting [8,39]. Psychological interventions have to focus on the reduction of maladaptive perfectionistic tendencies (concern over mistakes, doubt about action) to decrease the constant state of anxiety [118], which may lead to a lower incidence of NSSI. Cognitive-behavioural therapy (CBT) has a positive effect on perfectionism intervention [118], and it is worth considering the introduction of mindfulness techniques in school classes, which are effective in the case of emotion dysregulation [118] and decrease the relationship between perfectionism and emotional distress symptoms as well as its relationship with NSSI engagement [8]. According to our results, increasing the importance of order and neatness is protective against NSSI and may help adolescents organise daily tasks, which can lead to the sense that they can control and manage everyday situations [104]. Healthy perfectionistic students believe that teachers with a demand for organisation and neatness in schoolwork help them to organise their daily lives [119]. Mental health prevention school programs [70,100,101] are essential because the recognition of maladaptive perfectionistic tendencies is problematic for parents and teachers [119], and almost one-third of gifted adolescents have high levels of maladaptive perfectionist characteristics [119]. They do not seek help, try to hide their feelings and problems [115], and are unable to recognize the negative consequences of their continuous concern and self-criticism [119]. Maladaptive perfectionists often perceive high parental expectations and criticism [119], thus involving family members in intervention strategies would be beneficial. 

## 5. Limitations

Our findings need to be interpreted in light of the following limitations. Our evidence is based on a cross-sectional study design; that is why it does not provide information related to causality. NSSI was assessed as an outcome variable, and we examined its predictors and the association between them. A longitudinal study, focusing on a potential mediating effect, is required to provide evidence for a causal relationship between NSSI and maladaptive perfectionism. We used the MINI–KID interview for diagnostic assessment, and it contains questions only related to borderline personality disorder, and there are no questions regarding other forms of personality disorders. According to the instructions of the MINI–KID interview, the ASD diagnoses based on the MINI–KID should be investigated more thoroughly by a licensed child- and adolescent psychiatrist. This did not happen in our study. Related to the reliability assessment, MINI–KID symptoms were assessed with the KR-20 formula [77], which showed a lower reliability value (under 0.5) for mood, tic, and eating disorders in our study. We used self-report questionnaires for the assessment of perfectionism and NSSI. Our study should be considered preliminary because of the small sample size and the fact that the sample may have been biased towards more severe mental health concerns given that the participants came from classes in which teachers were concerned about the mental health of their students, which constricts the generalisability of the results to a wider population. 

## 6. Conclusions

Our study draws attention to an increasing trend and the extremely high NSSI prevalence rate among a Hungarian community adolescent sample, which must be considered with special attention. Adolescents with perfectionistic concerns are at a heightened risk of anxiety disorders, which can increase their vulnerability to NSSI engagement. The findings of this study emphasise the importance of targeted prevention and treatment related to NSSI engagement and effective interventions for maladaptive perfectionism, including the reduction of extremely high standards and the setting of achievable goals, which may decrease the risk of NSSI [39]. 

## Figures and Tables

**Table 1:** Prevalence rates of NSSI methods by gender.

Types of NSSI	NSSI	Male (*n* = 42)	Male %	Female (*n* = 104)	Female %	X^2^ (*p*-Value)
Cutting	No	35	83.33%	63	60.58%	15.04 (0.52)
	Yes	7	16.67%	41	39.42%	
Biting	No	30	71.43%	81	77.88%	11.80 (0.46)
	Yes	12	28.57%	23	22.12%	
Burning	No	35	83.33%	87	83.65%	6.06 (0.64)
	Yes	7	16.67%	17	16.35%	
Carving	No	34	80.95%	84	80.77%	6.92 (0.54)
	Yes	8	19.05%	20	19.23%	
Pinching	No	31	73.81%	88	84.62%	8.67 (0.56)
	Yes	11	26.19%	16	15.38%	
Hair pulling	No	35	83.33%	96	92.31%	6.24 (0.40)
	Yes	7	16.67%	8	7.69%	
Severe scratching	No	38	90.48%	86	82.69%	7.50 (0.68)
	Yes	4	9.52%	18	17.31%	
Banging/hitting	No	27	64.29%	77	74.04%	13.55 (0.63)
	Yes	15	35.71%	27	25.96%	
Wound picking	No	26	61.90%	73	70.19%	29.21 (0.11)
	Yes	16	38.10%	31	29.81%	
Rubbing skin against rough surfaces	No	37	88.10%	94	90.38%	4.50 (0.61)
	Yes	5	11.90%	10	9.62%	
Needle-sticking	No	40	95.24%	90	86.54%	7.06 (0.53)
	Yes	2	4.76%	14	13.46%	
Swallowing chemicals	No	37	88.10%	99	95.19%	7.72 (0.10)
	Yes	5	11.90%	5	4.81%	

Note. NSSI = nonsuicidal self-injury. X^2^—chi-square test, *p*-value—level of significance.

**Table 2:** Prevalence rates of mental disorders among adolescents who did and did not engage in NSSI.

Mental Disorder	*N* = 146	%	NSSI (*n* = 90)	%	No NSSI (*n* = 56)	%
Autism spectrum disorder ^a^	70	47.95	47	52.22	23	41.07
Suicidality	44	30.14	33	36.67	11	19.64
Mood disorders	41	28.08	33	36.67	8	14.29
Anxiety disorders	40	27.40	35	38.89	5	8.93
Substance use disorders	25	17.12	19	21.11	6	10.71
Eating disorders	21	14.38	19	21.11	2	3.57
Borderline personality disorder	16	10.96	14	15.56	2	3.57
Tic	14	9.59	10	11.11	4	7.14
Psychotic disorders	11	7.53	8	8.89	3	5.36
Attention-disruptive disorders	9	6.16	6	6.67	3	5.36

Note. NSSI = adolescents who engaged in nonsuicidal self-injury (NSSI); No NSSI = adolescents who did not engage in NSSI. ^a^ This means that on the basis of answers related to the questions on the Mini International Neuropsychiatric Interview Kid, a diagnosis of autism spectrum disorder could not be ruled out.

**Table 3:** Associations between perfectionism dimensions and NSSI engagement.

Outcome Variables	NSSI (*N* = 146)					NSSI Intrapersonal Motivation (*n* = 134)					NSSI Interpersonal Motivation (*n* = 134)				
	Estimate	SE	*t*	df	Pr (>|t|)	Estimate	SE	*t*	df	Pr (>|t|)	Estimate	SE	*t*	df	Pr (>|t|)
FMPS–CMD	0.04	0.02	1.70	141	0.09	0.05	0.02	2.94	128	<0.01 **	0.04	0.01	2.58	128	0.01 *
FMPS–PEC	−0.01	0.03	−0.46	141	0.65	<0.01	0.02	0.02	128	0.98	<0.01	0.02	−0.09	128	0.93
FMPS–PS	0.01	0.05	0.29	141	0.77	<0.01	0.04	−0.1	128	0.95	<0.01	0.03	−0.03	128	0.98
FMPS–O	−0.11	0.05	−2.07	141	0.04 *	−0.06	0.04	−1.7	128	0.1	−0.09	0.03	−2.82	128	0.01 *
Intercept	1.56	1.12	1.39	141	0.17	1.06	0.75	1.41	128	0.16	2.07	0.65	3.18	128	<0.01 **

Note. FMPS = Frost Multidimensional Perfectionism Scale; CMD = Concern Over Mistakes and Doubts About Actions subscales; PEC = Parental Expectations and Criticism subscales; PS = Personal Standards subscale; O = Organisation subscale; NSSI = nonsuicidal self-injury. SE—standard error, *t*-Value—*t*-tests, df—degrees of freedom, *p*—level of significance. * *p* < 0.05. ** *p* < 0.01.

**Table 4:** Associations between perfectionism dimensions and NSSI engagement after controlling for the effect of mental disorders.

Outcome Variable	NSSI (*N* = 146)					NSSI Intrapersonal Motivation (*n* = 134)					NSSI Interpersonal Motivation (*n* = 134)				
	Estimate	SE	*t*	df	Pr (>|t|)	Estimate	SE	*t*	df	Pr (>|t|)	Estimate	SE	*t*	df	Pr (>|t|)
Intercept	2.73	1.36	2	130	0.05 *	1.84	0.73	2.54	118	0.01 *	2.15	0.67	3.20	118	<0.01 **
Mood	0.88	0.62	1.43	130	0.16	0.63	0.34	1.83	118	0.07	0.02	0.33	0.05	118	0.96
Anxiety disorder	2.39	0.77	3.09	130	<0.01 **	1.03	0.37	2.79	118	<0.01 **	1.13	0.35	3.21	118	<0.01 **
Substance use disorder	0.91	0.65	1.4	130	0.17	0.30	0.37	0.81	118	0.42	0.32	0.34	0.94	118	0.35
Attention-disruptive disorders	−1.45	1.06	−1.38	130	0.17	−0.31	0.55	−0.56	118	0.58	−0.23	0.52	−0.45	118	0.65
Tic	0.44	0.79	0.56	130	0.58	−0.40	0.54	−0.74	118	0.46	0.24	0.46	0.51	118	0.61
Eating disorders	1.85	1	1.85	130	0.07	0.72	0.38	1.89	118	0.06	0.58	0.37	1.56	118	0.12
Psychotic disorders	−1.56	1.07	−1.45	130	0.15	−0.31	0.52	−0.60	118	0.55	−0.15	0.49	−0.31	118	0.75
Autism spectrum disorders	0.01	0.5	0.01	130	0.99	−0.11	0.33	−0.34	118	0.74	−0.05	0.31	−0.17	118	0.87
Borderline personality disorders	1.73	1.1	1.56	130	0.12	0.96	0.51	1.89	118	0.06	0.66	0.48	1.38	118	0.17
Suicidality	−0.27	0.63	−0.44	130	0.66	0.06	0.39	0.16	118	0.88	0.08	0.36	0.21	118	0.83
FMPS–CMD	<0.01	0.03	−0.05	130	0.96	0.02	0.02	0.99	118	0.33	0.01	0.02	0.63	118	0.53
FMPS–PEC	−0.07	0.04	−1.83	130	0.07	−0.03	0.02	−1.29	118	0.20	−0.02	0.02	−0.92	118	0.36
FMPS–PS	0.07	0.06	1.15	130	0.25	0.01	0.04	0.15	118	0.88	0.02	0.03	0.52	118	0.60
FMPS–O	−0.14	0.06	−2.36	130	0.02 *	−0.08	0.04	−2.13	118	0.04 *	−0.09	0.03	−2.75	118	<0.01 **

Note. FMPS = Frost Multidimensional Perfectionism Scale; CMD = Concern Over Mistakes and Doubts About Actions subscales; PEC = Parental Expectations and Criticism subscales; PS = Personal Standards subscale; O = Organisation subscale; NSSI = nonsuicidal self-injury. SE—standard error, *t*-Value—*t*-tests, df—degrees of freedom, *p*—level of significance. * *p* < 0.05. ** *p* < 0.01.

**Table 5:** Associations between perfectionism dimensions and anxiety disorders.

Outcome Variable	Anxiety Disorders				
	Estimate	SE	*t*	df	Pr (>|t|)
Intercept	−4.72	1.73	−2.73	140	0.01 **
FMPS–CMD	0.09	0.03	3.07	140	<0.01 **
FMPS–PEC	0.06	0.03	1.83	140	0.07
FMPS–PS	−0.08	0.07	−1.15	140	0.25
FMPS–O	0.03	0.07	0.47	140	0.64

Note. FMPS = Frost Multidimensional Perfectionism Scale; CMD = Concern Over Mistakes and Doubts About Actions subscales; PEC = Parental Expectations and Criticism subscales; PS = Personal Standards subscale; O = Organisation subscale; NSSI = nonsuicidal self-injury. SE—standard error, *t*-Value—*t*-tests, df—degrees of freedom, *p*—level of significance. ** *p* < 0.01.

**Table 6:** Associations between the perfectionism dimensions.

Variable	FMPS–PS		FMPS–CMD		FMPS–PEC	
	ρ	*p*	ρ	*p*	ρ	*p*
FMPS–CMD	0.68 ***	<0.001				
FMPS–PEC	0.37 ***	<0.001	0.49 ***	<0.001		
FMPS–O	0.45 ***	<0.001	0.16	0.05	0.11	0.17

Note. CMD = Concern Over Mistakes and Doubts About Actions subscales; FMPS = Frost Multidimensional Perfectionism Scale; O = Organisation subscale; PEC = Parental Expectations and Criticism subscales; PS = Personal Standards subscale. ρ—Spearman rho correlation, *p*—level of significance, *** *p* < 0.001.

## Data Availability

The raw data presented in this study are available on request from the corresponding author to any qualified researcher without undue reservation.

## Appendix A

The appendix contains numeric data related to network analysis.

**Table A1:** Numeric data related to Figure 1. Associations among NSSI, FMPS dimensions, and mental disorders.

Variable	Variable 1	Variable 2	Value	prop0	q2.5	q97.5	q2.5_non0	q97.5_non0
FMPS_CMD—FMPS_PS	FMPS_CMD	FMPS_PS	0.49	<0.01	0.38	0.58	0.38	0.58
FMPS_PS—FMPS_O	FMPS_PS	FMPS_O	0.36	<0.01	0.22	0.48	0.22	0.48
Anxiety—Suicid	Anxiety	Suicid	0.82	0.07	<0.01	1.60	0.28	1.62
BPD—Suicid	BPD	Suicid	4.23	0.14	<0.01	37.02	0.37	40.29
Mood—Suicid	Mood	Suicid	0.45	0.19	<0.01	1.02	0.21	1.06
Anxiety—NSSI	Anxiety	NSSI	0.49	0.29	<0.01	1.61	0.23	1.76
ASD—BPD	ASD	BPD	2.84	0.41	<0.01	28.78	0.34	31.78
FMPS_CMD—FMPS_PEC	FMPS_CMD	FMPS_PEC	0.13	0.43	<0.01	0.32	0.14	0.34
Anxiety—ASD	Anxiety	ASD	0.38	0.44	<0.01	1.22	0.21	1.39
Psychotic—BPD	Psychotic	BPD	1.98	0.44	<0.01	20.43	0.32	24.71
Atten_disr—BPD	Atten_disr	BPD	2.59	0.45	<0.01	23.83	0.41	29.20
Anxiety—FMPS_CMD	Anxiety	FMPS_CMD	0.21	0.48	<0.01	0.65	0.15	0.94
Mood—Anxiety	Mood	Anxiety	0.20	0.58	<0.01	0.76	0.17	0.85
ED—FMPS_CMD	ED	FMPS_CMD	0.23	0.59	<0.01	0.54	0.12	0.87
ED—Suicid	ED	Suicid	0.24	0.68	<0.01	0.84	0.21	1.20
Tic—Psychotic	Tic	Psychotic	0.62	0.68	<0.01	4.13	0.43	15.90
Mood—ED	Mood	ED	0.18	0.69	<0.01	0.71	0.17	0.89
Psychotic—Suicid	Psychotic	Suicid	0.65	0.71	<0.01	5.23	0.27	21.95
Anxiety—Psychotic	Anxiety	Psychotic	0.73	0.71	<0.01	3.04	0.26	31.76
Anxiety—ED	Anxiety	ED	0.25	0.72	<0.01	0.99	0.19	7.67
ED—BPD	ED	BPD	0.87	0.74	<0.01	10.14	0.25	27.91
Tic—ASD	Tic	ASD	0.37	0.74	<0.01	1.20	0.30	12.55
ASD—FMPS_O	ASD	FMPS_O	−0.06	0.76	−0.38	<0.01	−0.45	−0.15
Anxiety—FMPS_PEC	Anxiety	FMPS_PEC	0.07	0.77	<0.01	0.42	0.14	0.53
Mood—Psychotic	Mood	Psychotic	0.27	0.78	<0.01	1.49	0.22	7.86
ED—NSSI	ED	NSSI	0.18	0.79	<0.01	0.78	0.19	1.07
BPD—FMPS_CMD	BPD	FMPS_CMD	0.20	0.81	<0.01	2.53	0.18	7.58
ASD—FMPS_CMD	ASD	FMPS_CMD	0.04	0.84	<0.01	0.35	0.14	0.53
Atten_disr—Suicid	Atten_disr	Suicid	0.31	0.84	<0.01	2.72	0.30	15.99
Mood—Substance	Mood	Substance	0.06	0.87	<0.01	0.60	0.22	0.84
Mood—NSSI	Mood	NSSI	0.05	0.88	<0.01	0.46	0.14	0.94
Mood—Atten_disr	Mood	Atten_disr	0.15	0.90	<0.01	1.06	0.21	10.76
FMPS_PEC—FMPS_O	FMPS_PEC	FMPS_O	0.01	0.91	<0.01	0.14	0.07	0.20
Anxiety—Atten_disr	Anxiety	Atten_disr	0.16	0.91	<0.01	1.39	0.28	13.90
Anxiety—BPD	Anxiety	BPD	−0.74	0.91	−12.29	<0.01	−29.94	4.32
Tic—BPD	Tic	BPD	0.49	0.92	<0.01	3.92	0.26	47.45
BPD—NSSI	BPD	NSSI	0.68	0.92	<0.01	8.67	0.26	53.21
Anxiety—Substance	Anxiety	Substance	0.04	0.92	<0.01	0.53	0.19	0.97
Atten_disr—ASD	Atten_disr	ASD	0.29	0.93	<0.01	2.37	0.28	24.33
ASD—Suicid	ASD	Suicid	0.03	0.94	<0.01	0.45	0.16	0.93
Atten_disr—FMPS_PEC	Atten_disr	FMPS_PEC	0.05	0.94	<0.01	0.47	0.17	5.44
Substance—FMPS_PEC	Substance	FMPS_PEC	0.02	0.94	<0.01	0.26	0.10	0.45
ED—ASD	ED	ASD	−0.08	0.95	−0.63	<0.01	−20.18	0.39
Mood—FMPS_CMD	Mood	FMPS_CMD	0.01	0.96	<0.01	0.19	0.09	0.35
Substance—ASD	Substance	ASD	−0.02	0.96	−0.51	<0.01	−1.85	−0.24
Mood—FMPS_PEC	Mood	FMPS_PEC	0.01	0.96	<0.01	0.21	0.10	0.49
NSSI—FMPS_O	NSSI	FMPS_O	−0.01	0.96	−0.17	<0.01	−0.44	−0.10
Substance—Atten_disr	Substance	Atten_disr	0.05	0.97	<0.01	0.52	−0.98	12.60
ED—FMPS_PEC	ED	FMPS_PEC	0.01	0.97	<0.01	0.19	−0.40	1.03
Substance—BPD	Substance	BPD	0.16	0.97	<0.01	0.52	0.28	49.07
Substance—ED	Substance	ED	0.05	0.97	<0.01	0.28	0.22	37.25
Atten_disr—Psychotic	Atten_disr	Psychotic	<0.01	0.97	<0.01	0.32	−17.63	4.15
Psychotic—FMPS_CMD	Psychotic	FMPS_CMD	0.04	0.97	<0.01	0.19	0.14	14.80
FMPS_PEC—FMPS_PS	FMPS_PEC	FMPS_PS	<0.01	0.97	<0.01	0.11	0.09	0.25
Mood—BPD	Mood	BPD	0.04	0.97	<0.01	0.13	−1.23	8.30
Substance—NSSI	Substance	NSSI	0.01	0.97	<0.01	0.23	0.22	0.89
Atten_disr—ED	Atten_disr	ED	<0.01	0.98	<0.01	<0.01	−22.10	6.21
Tic—Suicid	Tic	Suicid	−0.13	0.98	<0.01	<0.01	−38.17	−0.37
Mood—Tic	Mood	Tic	0.05	0.98	<0.01	<0.01	0.35	26.08
Suicid—NSSI	Suicid	NSSI	<0.01	0.98	<0.01	<0.01	−1.02	1.00
Mood—ASD	Mood	ASD	−0.01	0.98	<0.01	<0.01	−1.63	0.26
NSSI—FMPS_CMD	NSSI	FMPS_CMD	<0.01	0.98	<0.01	<0.01	0.12	0.27
ED—Psychotic	ED	Psychotic	0.04	0.98	<0.01	<0.01	−3.87	42.14
Mood—FMPS_O	Mood	FMPS_O	<0.01	0.98	<0.01	<0.01	−0.30	−0.12
Psychotic—FMPS_O	Psychotic	FMPS_O	−0.03	0.98	<0.01	<0.01	−11.18	−0.19
Tic—FMPS_O	Tic	FMPS_O	<0.01	0.98	<0.01	<0.01	−0.73	−0.16
Psychotic—ASD	Psychotic	ASD	−0.03	0.99	<0.01	<0.01	−16.12	1.98
ASD—FMPS_PEC	ASD	FMPS_PEC	<0.01	0.99	<0.01	<0.01	0.11	0.37
Tic—FMPS_PEC	Tic	FMPS_PEC	−0.01	0.99	<0.01	<0.01	−2.31	−0.15
Suicid—FMPS_CMD	Suicid	FMPS_CMD	<0.01	0.99	<0.01	<0.01	0.09	0.55
Atten_disr—Tic	Atten_disr	Tic	−0.06	0.99	<0.01	<0.01	−52.09	0.71
ASD—NSSI	ASD	NSSI	<0.01	0.99	<0.01	<0.01	−0.41	0.56
Anxiety—Tic	Anxiety	Tic	0.01	0.99	<0.01	<0.01	−0.37	6.12
Substance—Psychotic	Substance	Psychotic	<0.01	0.99	<0.01	<0.01	−0.97	1.97
Tic—NSSI	Tic	NSSI	0.03	1.00	<0.01	<0.01	−2.50	18.05
Anxiety—FMPS_PS	Anxiety	FMPS_PS	<0.01	1.00	<0.01	<0.01	−0.83	−0.35
Anxiety—FMPS_O	Anxiety	FMPS_O	<0.01	1.00	<0.01	<0.01	−0.28	0.81
Atten_disr—NSSI	Atten_disr	NSSI	−0.05	1.00	<0.01	<0.01	−32.97	−1.63
BPD—FMPS_O	BPD	FMPS_O	−0.02	1.00	<0.01	<0.01	−9.99	−1.86
Tic—ED	Tic	ED	0.01	1.00	<0.01	<0.01	0.23	10.70
Substance—Suicid	Substance	Suicid	<0.01	1.00	<0.01	<0.01	0.25	0.50
Suicid—FMPS_PS	Suicid	FMPS_PS	<0.01	1.00	<0.01	<0.01	−0.47	−0.29
ASD—FMPS_PS	ASD	FMPS_PS	<0.01	1.00	<0.01	<0.01	−0.48	0.15
Atten_disr—FMPS_CMD	Atten_disr	FMPS_CMD	<0.01	1.00	<0.01	<0.01	−3.73	−0.81
BPD—FMPS_PEC	BPD	FMPS_PEC	<0.01	1.00	<0.01	<0.01	−2.42	−0.48
Psychotic—FMPS_PEC	Psychotic	FMPS_PEC	<0.01	1.00	<0.01	<0.01	−0.56	−0.35
ED—FMPS_O	ED	FMPS_O	<0.01	1.00	<0.01	<0.01	0.20	0.39
Suicid—FMPS_PEC	Suicid	FMPS_PEC	<0.01	1.00	<0.01	<0.01	0.17	0.36
Psychotic—NSSI	Psychotic	NSSI	<0.01	1.00	<0.01	<0.01	−3.28	−3.28
Substance—Tic	Substance	Tic	<0.01	1.00	<0.01	<0.01	−0.47	−0.47
Substance—FMPS_CMD	Substance	FMPS_CMD	<0.01	1.00	<0.01	<0.01	0.32	0.32
Suicid—FMPS_O	Suicid	FMPS_O	<0.01	1.00	<0.01	<0.01	−0.30	−0.30
NSSI—FMPS_PEC	NSSI	FMPS_PEC	<0.01	1.00	<0.01	<0.01	−0.28	−0.28
ED—FMPS_PS	ED	FMPS_PS	<0.01	1.00	<0.01	<0.01	0.27	0.27
NSSI—FMPS_PS	NSSI	FMPS_PS	<0.01	1.00	<0.01	<0.01	0.22	0.22
Atten_disr—FMPS_O	Atten_disr	FMPS_O	<0.01	1.00	<0.01	<0.01	<0.01	<0.01
Atten_disr—FMPS_PS	Atten_disr	FMPS_PS	<0.01	1.00	<0.01	<0.01	<0.01	<0.01
BPD—FMPS_PS	BPD	FMPS_PS	<0.01	1.00	<0.01	<0.01	<0.01	<0.01
FMPS_CMD—FMPS_O	FMPS_CMD	FMPS_O	<0.01	1.00	<0.01	<0.01	<0.01	<0.01
Mood—FMPS_PS	Mood	FMPS_PS	<0.01	1.00	<0.01	<0.01	<0.01	<0.01
Psychotic—FMPS_PS	Psychotic	FMPS_PS	<0.01	1.00	<0.01	<0.01	<0.01	<0.01
Substance—FMPS_O	Substance	FMPS_O	<0.01	1.00	<0.01	<0.01	<0.01	<0.01
Substance—FMPS_PS	Substance	FMPS_PS	<0.01	1.00	<0.01	<0.01	<0.01	<0.01
Tic—FMPS_CMD	Tic	FMPS_CMD	<0.01	1.00	<0.01	<0.01	<0.01	<0.01
Tic—FMPS_PS	Tic	FMPS_PS	<0.01	1.00	<0.01	<0.01	<0.01	<0.01

Note: The value column refers to the point estimate of a given edge. The prop0 column demonstrates the proportion of bootstraps that resulted in zero edge weight. Columns q2.5 and q97.5 contain the lower and upper bounds of the bootstrapped confidence intervals after the regularization. These are calculated by ranking the 1000 bootstrapped weights for a given edge from the smallest to the largest and selecting the 25th and 975th values. Finally, q2.5non0 and q97.5non0 show the bootstrapped confidence interval calculated in the same way but only for the non-zero values. Anxiety = anxiety disorders; ASD = autism spectrum disorder; Atten_disr = attention-disruptive disorders; BPD = borderline personality disorders; CMD = Concern Over Mistakes and Doubts About Actions subscales; ED = eating disorders; FMPS = Frost Multidimensional Perfectionism Scale; Mood = mood disorders; NSSI = nonsuicidal self-injury; O = Organisation subscale; PEC = Parental Expectations and Criticism subscales; PS = Personal Standards subscale; Psychotic = psychotic disorders; Substance = substance use disorders; Suicid = suicidality.

**Table A2:** Numeric data related to Figure 2. Associations among NSSI intrapersonal function, FMPS dimensions, and mental disorders.

Variable	Variable 1	Variable 2	Value	prop0	q2.5	q97.5	q2.5_non0	q97.5_non0
Anxiety—Atten_disr	Anxiety	Atten_disr	0.16	0.90	<0.01	0.79	0.25	18.67
Anxiety—ASD	Anxiety	ASD	0.26	0.63	<0.01	1.25	0.21	2.17
Anxiety—BPD	Anxiety	BPD	−0.91	0.89	−12.67	<0.01	−36.68	2.34
Anxiety—ED	Anxiety	ED	0.34	0.75	<0.01	1.21	0.17	14.88
Anxiety—FMPS_CMD	Anxiety	FMPS_CMD	0.15	0.60	<0.01	0.50	0.14	0.65
Anxiety—FMPS_O	Anxiety	FMPS_O	<0.01	1.00	<0.01	<0.01	0.28	1.88
Anxiety—FMPS_PEC	Anxiety	FMPS_PEC	0.09	0.70	<0.01	0.46	0.16	0.57
Anxiety—FMPS_PS	Anxiety	FMPS_PS	<0.01	1.00	<0.01	<0.01	<0.01	<0.01
Anxiety—NSSI_intra	Anxiety	NSSI_intra	0.76	0.12	<0.01	1.58	0.25	1.67
Anxiety—Psychotic	Anxiety	Psychotic	0.83	0.68	<0.01	7.19	0.26	22.18
Anxiety—Substance	Anxiety	Substance	0.02	0.96	<0.01	0.45	0.16	1.19
Anxiety—Suicid	Anxiety	Suicid	1.07	0.05	<0.01	2.68	0.32	2.70
Anxiety—Tic	Anxiety	Tic	−0.05	1.00	<0.01	<0.01	−44.40	−8.14
Atten_disr—ASD	Atten_disr	ASD	0.20	0.94	<0.01	1.26	0.23	22.30
Atten_disr—BPD	Atten_disr	BPD	1.79	0.54	<0.01	15.35	0.43	24.66
Atten_disr—ED	Atten_disr	ED	0.10	0.97	<0.01	0.43	−3.61	50.86
Atten_disr—FMPS_CMD	Atten_disr	FMPS_CMD	<0.01	1.00	<0.01	<0.01	0.25	0.25
Atten_disr—FMPS_O	Atten_disr	FMPS_O	<0.01	1.00	<0.01	<0.01	<0.01	<0.01
Atten_disr—FMPS_PEC	Atten_disr	FMPS_PEC	0.03	0.94	<0.01	0.46	0.20	4.45
Atten_disr—FMPS_PS	Atten_disr	FMPS_PS	<0.01	1.00	<0.01	<0.01	<0.01	<0.01
Atten_disr—NSSI_intra	Atten_disr	NSSI_intra	<0.01	1.00	<0.01	<0.01	−1.42	−1.42
Atten_disr—Psychotic	Atten_disr	Psychotic	0.03	0.98	<0.01	<0.01	−11.64	13.81
Atten_disr—Suicid	Atten_disr	Suicid	0.20	0.88	<0.01	1.44	0.27	10.17
Atten_disr—Tic	Atten_disr	Tic	−0.01	0.99	<0.01	<0.01	−21.38	13.62
ASD—BPD	ASD	BPD	4.15	0.35	<0.01	32.82	0.33	36.56
ASD—FMPS_CMD	ASD	FMPS_CMD	0.05	0.83	<0.01	0.38	0.12	0.62
ASD—FMPS_O	ASD	FMPS_O	−0.04	0.86	−0.37	<0.01	−0.53	−0.14
ASD—FMPS_PEC	ASD	FMPS_PEC	<0.01	0.98	<0.01	<0.01	0.10	0.38
ASD—FMPS_PS	ASD	FMPS_PS	<0.01	1.00	<0.01	<0.01	−0.35	−0.27
ASD—NSSI_intra	ASD	NSSI_intra	<0.01	0.99	<0.01	<0.01	−0.62	0.82
ASD—Suicid	ASD	Suicid	−0.05	0.97	<0.01	<0.01	−46.84	2.07
BPD—FMPS_CMD	BPD	FMPS_CMD	0.10	0.88	<0.01	0.75	0.18	5.04
BPD—FMPS_O	BPD	FMPS_O	<0.01	1.00	<0.01	<0.01	−4.41	−4.41
BPD—FMPS_PEC	BPD	FMPS_PEC	−0.02	0.99	<0.01	<0.01	−2.98	−0.40
BPD—FMPS_PS	BPD	FMPS_PS	<0.01	1.00	<0.01	<0.01	<0.01	<0.01
BPD—NSSI_intra	BPD	NSSI_intra	2.23	0.58	<0.01	19.10	0.30	26.05
BPD—Suicid	BPD	Suicid	5.50	0.16	<0.01	41.77	0.39	43.17
ED—ASD	ED	ASD	−0.43	0.94	−1.35	<0.01	−60.63	−0.30
ED—BPD	ED	BPD	0.95	0.74	<0.01	12.33	0.27	32.50
ED—FMPS_CMD	ED	FMPS_CMD	0.30	0.76	<0.01	0.88	0.14	13.52
ED—FMPS_O	ED	FMPS_O	<0.01	1.00	<0.01	<0.01	0.11	0.11
ED—FMPS_PEC	ED	FMPS_PEC	0.02	0.96	<0.01	0.22	0.11	7.27
ED—FMPS_PS	ED	FMPS_PS	<0.01	1.00	<0.01	<0.01	<0.01	<0.01
ED—NSSI_intra	ED	NSSI_intra	0.89	0.32	<0.01	5.71	0.24	9.82
ED—Psychotic	ED	Psychotic	−0.17	0.98	<0.01	<0.01	−97.13	31.82
ED—Suicid	ED	Suicid	0.26	0.80	<0.01	0.97	0.23	17.47
FMPS_CMD—FMPS_O	FMPS_CMD	FMPS_O	<0.01	1.00	<0.01	<0.01	<0.01	<0.01
FMPS_CMD—FMPS_PEC	FMPS_CMD	FMPS_PEC	0.14	0.41	<0.01	0.33	0.14	0.35
FMPS_CMD—FMPS_PS	FMPS_CMD	FMPS_PS	0.49	<0.01	0.38	0.59	0.38	0.59
FMPS_PEC—FMPS_O	FMPS_PEC	FMPS_O	0.01	0.91	<0.01	0.15	0.08	0.21
FMPS_PEC—FMPS_PS	FMPS_PEC	FMPS_PS	<0.01	0.98	<0.01	<0.01	0.09	0.21
FMPS_PS—FMPS_O	FMPS_PS	FMPS_O	0.35	<0.01	0.21	0.49	0.22	0.49
Mood—Anxiety	Mood	Anxiety	0.08	0.84	<0.01	0.50	0.14	1.51
Mood—Atten_disr	Mood	Atten_disr	0.13	0.89	<0.01	0.89	0.25	10.88
Mood—ASD	Mood	ASD	<0.01	0.99	<0.01	<0.01	−1.00	0.89
Mood—BPD	Mood	BPD	0.09	0.97	<0.01	0.50	−0.38	17.36
Mood—ED	Mood	ED	0.27	0.68	<0.01	0.75	0.17	1.02
Mood—FMPS_CMD	Mood	FMPS_CMD	0.01	0.96	<0.01	0.17	0.11	0.35
Mood—FMPS_O	Mood	FMPS_O	<0.01	0.99	<0.01	<0.01	−0.38	−0.12
Mood—FMPS_PEC	Mood	FMPS_PEC	0.01	0.94	<0.01	0.26	0.08	0.40
Mood—FMPS_PS	Mood	FMPS_PS	<0.01	1.00	<0.01	<0.01	<0.01	<0.01
Mood—NSSI_intra	Mood	NSSI_intra	0.08	0.76	<0.01	0.51	0.16	0.65
Mood—Psychotic	Mood	Psychotic	0.33	0.75	<0.01	1.71	0.22	11.56
Mood—Substance	Mood	Substance	0.01	0.98	<0.01	<0.01	0.19	1.57
Mood—Suicid	Mood	Suicid	0.52	0.15	<0.01	1.14	0.25	1.16
Mood—Tic	Mood	Tic	<0.01	0.99	<0.01	<0.01	0.34	0.93
NSSI_intra—FMPS_CMD	NSSI_intra	FMPS_CMD	0.01	0.95	<0.01	0.20	0.11	0.66
NSSI_intra—FMPS_O	NSSI_intra	FMPS_O	−0.01	0.94	−0.25	<0.01	−0.47	−0.11
NSSI_intra—FMPS_PEC	NSSI_intra	FMPS_PEC	<0.01	0.99	<0.01	<0.01	−0.36	0.19
NSSI_intra—FMPS_PS	NSSI_intra	FMPS_PS	<0.01	1.00	<0.01	<0.01	<0.01	<0.01
Psychotic—ASD	Psychotic	ASD	−0.07	0.97	<0.01	<0.01	−56.08	16.12
Psychotic—BPD	Psychotic	BPD	1.56	0.54	<0.01	14.07	0.34	20.52
Psychotic—FMPS_CMD	Psychotic	FMPS_CMD	0.04	0.97	<0.01	0.27	0.16	10.82
Psychotic—FMPS_O	Psychotic	FMPS_O	−0.04	0.98	<0.01	<0.01	−19.63	−0.27
Psychotic—FMPS_PEC	Psychotic	FMPS_PEC	<0.01	1.00	<0.01	<0.01	<0.01	<0.01
Psychotic—FMPS_PS	Psychotic	FMPS_PS	<0.01	1.00	<0.01	<0.01	<0.01	<0.01
Psychotic—NSSI_intra	Psychotic	NSSI_intra	<0.01	0.99	<0.01	<0.01	−7.85	2.45
Psychotic—Suicid	Psychotic	Suicid	0.68	0.75	<0.01	2.60	0.24	30.18
Substance—Atten_disr	Substance	Atten_disr	0.04	0.95	<0.01	0.64	−2.07	7.42
Substance—ASD	Substance	ASD	−0.02	0.98	<0.01	<0.01	−4.00	−0.36
Substance—BPD	Substance	BPD	0.19	0.95	<0.01	2.06	0.44	20.39
Substance—ED	Substance	ED	0.01	0.99	<0.01	<0.01	0.34	0.68
Substance—FMPS_CMD	Substance	FMPS_CMD	<0.01	1.00	<0.01	<0.01	0.26	0.26
Substance—FMPS_O	Substance	FMPS_O	<0.01	1.00	<0.01	<0.01	<0.01	<0.01
Substance—FMPS_PEC	Substance	FMPS_PEC	0.03	0.90	<0.01	0.33	0.13	0.45
Substance—FMPS_PS	Substance	FMPS_PS	<0.01	1.00	<0.01	<0.01	<0.01	<0.01
Substance—NSSI_intra	Substance	NSSI_intra	0.01	0.98	<0.01	<0.01	0.19	0.68
Substance—Psychotic	Substance	Psychotic	−0.04	1.00	<0.01	<0.01	−32.19	0.72
Substance—Suicid	Substance	Suicid	<0.01	1.00	<0.01	<0.01	−0.44	0.79
Substance—Tic	Substance	Tic	<0.01	1.00	<0.01	<0.01	<0.01	<0.01
Suicid—FMPS_CMD	Suicid	FMPS_CMD	0.01	0.98	<0.01	<0.01	0.14	7.34
Suicid—FMPS_O	Suicid	FMPS_O	<0.01	1.00	<0.01	<0.01	−0.35	−0.35
Suicid—FMPS_PEC	Suicid	FMPS_PEC	<0.01	1.00	<0.01	<0.01	<0.01	<0.01
Suicid—FMPS_PS	Suicid	FMPS_PS	<0.01	1.00	<0.01	<0.01	−1.08	−0.35
Suicid—NSSI_intra	Suicid	NSSI_intra	0.05	0.90	<0.01	0.46	0.17	1.75
Tic—ASD	Tic	ASD	0.42	0.78	<0.01	1.91	0.28	18.46
Tic—BPD	Tic	BPD	0.64	0.90	<0.01	8.80	0.33	36.51
Tic—ED	Tic	ED	0.09	0.99	<0.01	<0.01	−10.94	40.63
Tic—FMPS_CMD	Tic	FMPS_CMD	<0.01	1.00	<0.01	<0.01	<0.01	<0.01
Tic—FMPS_O	Tic	FMPS_O	<0.01	1.00	<0.01	<0.01	−0.32	−0.23
Tic—FMPS_PEC	Tic	FMPS_PEC	−0.03	0.99	<0.01	<0.01	−22.92	−0.16
Tic—FMPS_PS	Tic	FMPS_PS	<0.01	1.00	<0.01	<0.01	<0.01	<0.01
Tic—NSSI_intra	Tic	NSSI_intra	−0.03	1.00	<0.01	<0.01	−27.90	−0.52
Tic—Psychotic	Tic	Psychotic	1.00	0.68	<0.01	8.01	0.37	38.19
Tic—Suicid	Tic	Suicid	−0.16	0.98	<0.01	<0.01	−44.99	−0.37

Note: The value column refers to the point estimate of a given edge. The prop0 column demonstrates the proportion of bootstraps that resulted in zero edge weight. Columns q2.5 and q97.5 contain the lower and upper bounds of the bootstrapped confidence intervals after the regularization. These are calculated by ranking the 1000 bootstrapped weights for a given edge from the smallest to the largest and selecting the 25th and 975th values. Finally, q2.5non0 and q97.5non0 show the bootstrapped confidence interval calculated in the same way but only for the non-zero values. Anxiety = anxiety disorders; ASD = autism spectrum disorder; Atten_disr = attention-disruptive disorders; BPD = borderline personality disorders; CMD = Concern Over Mistakes and Doubts About Actions subscales; ED = eating disorders; FMPS = Frost Multidimensional Perfectionism Scale; Mood = mood disorders; NSSI_intra = intrapersonal motivation of nonsuicidal self-injury; O = Organisation subscale; PEC = Parental Expectations and Criticism subscales; PS = Personal Standards subscale; Psychotic = psychotic disorders; Substance = substance use disorders; Suicid = suicidality.

**Table A3:** Numeric data related to Figure 3. Associations among NSSI interpersonal function, FMPS dimensions, and mental disorders.

Variable	Variable 1	Variable 2	Value	prop0	q2.5	q97.5	q2.5_non0	q97.5_non0
FMPS_CMD—FMPS_PS	FMPS_CMD	FMPS_PS	0.49	<0.01	0.38	0.59	0.38	0.59
FMPS_PS—FMPS_O	FMPS_PS	FMPS_O	0.35	<0.01	0.19	0.48	0.19	0.48
Anxiety—Suicid	Anxiety	Suicid	1.41	0.04	<0.01	5.60	0.35	5.89
Anxiety—NSSI_inter	Anxiety	NSSI_inter	0.91	0.13	<0.01	2.16	0.27	3.09
Mood—Suicid	Mood	Suicid	0.57	0.13	<0.01	1.17	0.26	1.21
BPD—Suicid	BPD	Suicid	5.07	0.15	<0.01	40.04	0.39	43.35
ASD—BPD	ASD	BPD	4.14	0.35	<0.01	30.40	0.39	34.97
ED—NSSI_inter	ED	NSSI_inter	0.71	0.37	<0.01	3.08	0.24	7.61
FMPS_CMD—FMPS_PEC	FMPS_CMD	FMPS_PEC	0.14	0.39	<0.01	0.34	0.15	0.37
BPD—NSSI_inter	BPD	NSSI_inter	2.45	0.53	<0.01	19.85	0.29	26.04
Psychotic—BPD	Psychotic	BPD	1.55	0.54	<0.01	13.82	0.39	24.97
Atten_disr—BPD	Atten_disr	BPD	1.67	0.56	<0.01	16.00	0.44	27.76
Anxiety—FMPS_CMD	Anxiety	FMPS_CMD	0.26	0.56	<0.01	0.70	0.14	2.31
Anxiety—ASD	Anxiety	ASD	0.35	0.57	<0.01	1.34	0.22	2.67
Tic—Psychotic	Tic	Psychotic	0.84	0.65	<0.01	5.69	0.39	19.02
Mood—ED	Mood	ED	0.29	0.66	<0.01	0.85	0.20	4.02
Anxiety—Psychotic	Anxiety	Psychotic	0.81	0.69	<0.01	7.45	0.26	26.60
ED—FMPS_CMD	ED	FMPS_CMD	0.37	0.72	<0.01	2.63	0.14	14.57
Anxiety—FMPS_PEC	Anxiety	FMPS_PEC	0.11	0.73	<0.01	0.49	0.14	0.77
Mood—Psychotic	Mood	Psychotic	0.35	0.73	<0.01	1.46	0.23	10.44
Psychotic—Suicid	Psychotic	Suicid	0.46	0.74	<0.01	2.31	0.28	14.26
ED—Suicid	ED	Suicid	0.22	0.76	<0.01	1.00	0.20	5.52
Tic—ASD	Tic	ASD	0.54	0.77	<0.01	2.88	0.30	20.34
ED—BPD	ED	BPD	0.97	0.77	<0.01	10.60	0.27	34.20
Anxiety—ED	Anxiety	ED	0.38	0.79	<0.01	1.90	0.17	20.95
Mood—Anxiety	Mood	Anxiety	0.16	0.79	<0.01	0.62	0.17	2.91
ASD—FMPS_CMD	ASD	FMPS_CMD	0.06	0.80	<0.01	0.40	0.12	0.54
ASD—FMPS_O	ASD	FMPS_O	−0.05	0.82	−0.34	<0.01	−0.46	−0.13
BPD—FMPS_CMD	BPD	FMPS_CMD	0.19	0.85	<0.01	1.97	0.18	8.92
Atten_disr—Suicid	Atten_disr	Suicid	0.29	0.85	<0.01	2.57	0.30	15.59
Substance—FMPS_PEC	Substance	FMPS_PEC	0.03	0.88	<0.01	0.36	0.13	0.55
Mood—Atten_disr	Mood	Atten_disr	0.11	0.89	<0.01	0.80	0.26	6.01
Anxiety—BPD	Anxiety	BPD	−1.22	0.89	−15.38	<0.01	−59.78	−0.44
Tic—BPD	Tic	BPD	0.66	0.90	<0.01	7.87	0.47	32.39
Anxiety—Atten_disr	Anxiety	Atten_disr	0.15	0.92	<0.01	1.10	0.25	19.11
FMPS_PEC—FMPS_O	FMPS_PEC	FMPS_O	0.01	0.92	<0.01	0.15	0.07	0.22
Atten_disr—ASD	Atten_disr	ASD	0.26	0.93	<0.01	1.47	0.23	21.32
ED—ASD	ED	ASD	−0.33	0.94	−1.78	<0.01	−34.11	−0.32
Mood—FMPS_PEC	Mood	FMPS_PEC	0.01	0.94	<0.01	0.24	0.10	0.51
Substance—NSSI_inter	Substance	NSSI_inter	0.02	0.95	<0.01	0.40	0.16	0.87
Atten_disr—FMPS_PEC	Atten_disr	FMPS_PEC	0.03	0.95	<0.01	0.37	0.15	4.44
Substance—BPD	Substance	BPD	0.13	0.95	<0.01	1.09	0.35	16.46
ED—FMPS_PEC	ED	FMPS_PEC	−0.01	0.95	<0.01	0.29	−25.61	6.27
Anxiety—Substance	Anxiety	Substance	0.05	0.96	<0.01	0.47	0.29	13.36
Suicid—NSSI_inter	Suicid	NSSI_inter	0.01	0.96	<0.01	0.25	−1.93	3.74
Substance—Atten_disr	Substance	Atten_disr	0.04	0.96	<0.01	0.62	0.30	9.28
Mood—FMPS_CMD	Mood	FMPS_CMD	0.01	0.96	<0.01	0.18	0.09	0.34
Mood—BPD	Mood	BPD	0.07	0.96	<0.01	0.38	−2.85	24.49
Substance—ASD	Substance	ASD	−0.03	0.97	−0.55	<0.01	−5.83	−0.32
Psychotic—FMPS_CMD	Psychotic	FMPS_CMD	0.01	0.97	<0.01	0.20	0.15	1.47
NSSI_inter—FMPS_CMD	NSSI_inter	FMPS_CMD	0.01	0.97	<0.01	0.12	0.12	0.35
NSSI_inter—FMPS_PS	NSSI_inter	FMPS_PS	0.01	0.98	<0.01	<0.01	0.10	0.50
ASD—Suicid	ASD	Suicid	0.01	0.98	<0.01	<0.01	−2.33	2.12
Atten_disr—ED	Atten_disr	ED	0.05	0.98	<0.01	<0.01	0.27	18.67
ED—Psychotic	ED	Psychotic	−0.11	0.98	<0.01	<0.01	−46.25	10.41
ASD—FMPS_PEC	ASD	FMPS_PEC	0.01	0.98	<0.01	<0.01	0.09	0.42
Suicid—FMPS_CMD	Suicid	FMPS_CMD	0.01	0.98	<0.01	<0.01	0.11	3.42
Tic—Suicid	Tic	Suicid	−0.30	0.98	<0.01	<0.01	−120.68	−0.42
Mood—Substance	Mood	Substance	0.01	0.98	<0.01	<0.01	0.25	0.85
NSSI_inter—FMPS_O	NSSI_inter	FMPS_O	<0.01	0.98	<0.01	<0.01	−0.50	−0.14
Mood—NSSI_inter	Mood	NSSI_inter	<0.01	0.98	<0.01	<0.01	−1.00	0.57
FMPS_PEC—FMPS_PS	FMPS_PEC	FMPS_PS	<0.01	0.98	<0.01	<0.01	0.09	0.25
Tic—FMPS_PEC	Tic	FMPS_PEC	−0.02	0.98	<0.01	<0.01	−6.02	−0.16
Psychotic—FMPS_O	Psychotic	FMPS_O	−0.01	0.98	<0.01	<0.01	−0.95	−0.25
Atten_disr—Psychotic	Atten_disr	Psychotic	0.03	0.98	<0.01	<0.01	0.25	10.27
Psychotic—ASD	Psychotic	ASD	0.18	0.98	<0.01	<0.01	−1.98	85.01
Mood—ASD	Mood	ASD	−0.01	0.99	<0.01	<0.01	−1.45	0.45
Psychotic—NSSI_inter	Psychotic	NSSI_inter	<0.01	0.99	<0.01	<0.01	−15.19	23.88
ASD—NSSI_inter	ASD	NSSI_inter	<0.01	0.99	<0.01	<0.01	−0.44	0.63
Mood—FMPS_O	Mood	FMPS_O	<0.01	0.99	<0.01	<0.01	−0.29	−0.18
BPD—FMPS_PEC	BPD	FMPS_PEC	−0.01	0.99	<0.01	<0.01	−2.47	−0.30
Tic—ED	Tic	ED	0.01	0.99	<0.01	<0.01	−13.67	15.92
Substance—ED	Substance	ED	<0.01	0.99	<0.01	<0.01	0.13	0.71
Mood—Tic	Mood	Tic	<0.01	0.99	<0.01	<0.01	0.32	0.63
Tic—NSSI_inter	Tic	NSSI_inter	0.01	0.99	<0.01	<0.01	−0.31	3.66
Atten_disr—Tic	Atten_disr	Tic	0.09	0.99	<0.01	<0.01	−7.95	62.98
Anxiety—Tic	Anxiety	Tic	−0.03	1.00	<0.01	<0.01	−19.25	1.54
Substance—Suicid	Substance	Suicid	<0.01	1.00	<0.01	<0.01	−0.66	0.51
Substance—Psychotic	Substance	Psychotic	<0.01	1.00	<0.01	<0.01	−6.27	4.68
Suicid—FMPS_PS	Suicid	FMPS_PS	<0.01	1.00	<0.01	<0.01	−0.70	−0.34
ASD—FMPS_PS	ASD	FMPS_PS	<0.01	1.00	<0.01	<0.01	−0.86	−0.28
Suicid—FMPS_PEC	Suicid	FMPS_PEC	<0.01	1.00	<0.01	<0.01	−0.81	0.22
Atten_disr—NSSI_inter	Atten_disr	NSSI_inter	−0.04	1.00	<0.01	<0.01	−43.39	−1.54
BPD—FMPS_O	BPD	FMPS_O	<0.01	1.00	<0.01	<0.01	−3.28	−1.06
NSSI_inter—FMPS_PEC	NSSI_inter	FMPS_PEC	<0.01	1.00	<0.01	<0.01	−0.28	−0.18
Tic—FMPS_O	Tic	FMPS_O	<0.01	1.00	<0.01	<0.01	−0.25	−0.21
Substance—FMPS_CMD	Substance	FMPS_CMD	<0.01	1.00	<0.01	<0.01	−0.24	0.23
Psychotic—FMPS_PEC	Psychotic	FMPS_PEC	<0.01	1.00	<0.01	<0.01	0.17	0.75
Tic—FMPS_PS	Tic	FMPS_PS	<0.01	1.00	<0.01	<0.01	−3.02	−3.02
Anxiety—FMPS_PS	Anxiety	FMPS_PS	<0.01	1.00	<0.01	<0.01	−1.00	−1.00
Suicid—FMPS_O	Suicid	FMPS_O	<0.01	1.00	<0.01	<0.01	−0.24	−0.24
FMPS_CMD—FMPS_O	FMPS_CMD	FMPS_O	<0.01	1.00	<0.01	<0.01	−0.16	−0.16
Substance—FMPS_PS	Substance	FMPS_PS	<0.01	1.00	<0.01	<0.01	0.12	0.12
Atten_disr—FMPS_CMD	Atten_disr	FMPS_CMD	<0.01	1.00	<0.01	<0.01	0.20	0.20
Tic—FMPS_CMD	Tic	FMPS_CMD	0.01	1.00	<0.01	<0.01	7.19	7.19
Anxiety—FMPS_O	Anxiety	FMPS_O	<0.01	1.00	<0.01	<0.01	<0.01	<0.01
Atten_disr—FMPS_O	Atten_disr	FMPS_O	<0.01	1.00	<0.01	<0.01	<0.01	<0.01
Atten_disr—FMPS_PS	Atten_disr	FMPS_PS	<0.01	1.00	<0.01	<0.01	<0.01	<0.01
BPD—FMPS_PS	BPD	FMPS_PS	<0.01	1.00	<0.01	<0.01	<0.01	<0.01
ED—FMPS_O	ED	FMPS_O	<0.01	1.00	<0.01	<0.01	<0.01	<0.01
ED—FMPS_PS	ED	FMPS_PS	<0.01	1.00	<0.01	<0.01	<0.01	<0.01
Mood—FMPS_PS	Mood	FMPS_PS	<0.01	1.00	<0.01	<0.01	<0.01	<0.01
Psychotic—FMPS_PS	Psychotic	FMPS_PS	<0.01	1.00	<0.01	<0.01	<0.01	<0.01
Substance—FMPS_O	Substance	FMPS_O	<0.01	1.00	<0.01	<0.01	<0.01	<0.01
Substance—Tic	Substance	Tic	<0.01	1.00	<0.01	<0.01	<0.01	<0.01

Note: The value column refers to the point estimate of a given edge. The prop0 column demonstrates the proportion of bootstraps that resulted in zero edge weight. Columns q2.5 and q97.5 contain the lower and upper bound of the bootstrapped confidence intervals after the regularization. These are calculated by ranking the 1000 bootstrapped weights for a given edge from the smallest to the largest and selecting the 25th and 975th values. Finally, q2.5non0 and q97.5non0 show the bootstrapped confidence interval calculated in the same way but only for the non-zero values. Anxiety = anxiety disorders; ASD = autism spectrum disorder; Atten_disr = attention-disruptive disorders; BPD = borderline personality disorders; CMD = Concern Over Mistakes and Doubts About Actions subscales; ED = eating disorders; FMPS = Frost Multidimensional Perfectionism Scale; Mood = mood disorders; NSSI_inter = interpersonal motivation of nonsuicidal self-injury; O = Organisation subscale; PEC = Parental Expectations and Criticism subscales; PS = Personal Standards subscale; Psychotic = psychotic disorders; Substance = substance use disorders; Suicid = suicidality.

**Table A4:** Associations between perfectionism dimensions and NSSI functions (original ISAS categorisation).

Outcome Variables	NSSI Intrapersonal Motivation					NSSI Interpersonal Motivation				
	(*n* = 134)					(*n* = 134)				
	Estimate	SE	*t*	df	Pr (>|t|)	Estimate	SE	*t*	df	Pr (>|t|)
FMPS–CMD	0.04	0.02	2.00	128	0.05 *	0.04	0.02	1.88	128	0.06
FMPS–PEC	0.01	0.03	0.24	128	0.81	0.00	0.03	0.02	128	0.99
FMPS–PS	0.00	0.04	0.01	128	0.99	0.01	0.04	0.22	128	0.82
FMPS–O	−0.06	0.04	−1.40	128	0.16	−0.12	0.04	−2.74	128	0.01 *
Intercept	1.43	0.96	1.49	128	0.14	2.08	0.89	2.33	128	0.02 *

Note. FMPS = Frost Multidimensional Perfectionism Scale; CMD = Concern Over Mistakes and Doubts About Actions subscales; PEC = Parental Expectations and Criticism subscales; PS = Personal Standards subscale; O = Organisation subscale; NSSI = nonsuicidal self-injury. SE—standard error, *t*-Value—*t*-tests, df—degrees of freedom, *p*—level of significance. * *p* <0.05.

**Table A5:** Associations between perfectionism dimensions and NSSI functions (original ISAS categorisation) after controlling for the effect of mental disorders.

Outcome Variable	NSSI Intrapersonal Motivation					NSSI Interpersonal Motivation				
	(*n* = 134)					(*n* = 134)				
	Estimate	SE	*t*	df	Pr (>|t|)	Estimate	SE	*t*	df	Pr (>|t|)
Intercept	2.66	0.85	3.12	118	<0.01 **	2.34	0.82	2.84	118	<0.01 **
Mood	0.59	0.40	1.47	118	0.14	0.06	0.41	0.14	118	0.89
Anxiety disorder	1.29	0.44	2.97	118	<0.01 **	1.21	0.43	2.81	118	<0.01 **
Substance use disorder	0.29	0.42	0.69	118	0.49	0.45	0.42	1.09	118	0.28
Attention-disruptive disorders	−0.48	0.66	−0.73	118	0.47	−0.10	0.64	−0.16	118	0.88
Tic	−0.36	0.57	−0.63	118	0.53	0.47	0.54	0.87	118	0.38
Eating disorders	0.89	0.47	1.89	118	0.06	0.81	0.47	1.74	118	0.08
Psychotic disorders	−0.28	0.63	−0.45	118	0.65	−0.54	0.61	−0.88	118	0.38
Autism spectrum disorders	−0.11	0.36	−0.29	118	0.77	0.01	0.36	0.03	118	0.98
Borderline personality disorders	1.05	0.60	1.76	118	0.08	1.11	0.59	1.88	118	0.06
Suicidality	−0.04	0.44	−0.09	118	0.93	−0.27	0.44	−0.61	118	0.54
FMPS–CMD	0.01	0.02	0.51	118	0.61	0.00	0.02	0.19	118	0.85
FMPS–PEC	−0.04	0.02	−1.50	118	0.13	−0.01	0.02	−0.28	118	0.78
FMPS–PS	0.01	0.04	0.17	118	0.87	0.03	0.04	0.84	118	0.4
FMPS–O	−0.09	0.04	−2.23	118	0.03 *	−0.13	0.04	−3.35	118	<0.01 **

Note. FMPS = Frost Multidimensional Perfectionism Scale; CMD = Concern Over Mistakes and Doubts About Actions subscales; PEC = Parental Expectations and Criticism subscales; PS = Personal Standards subscale; O = Organisation subscale; NSSI = nonsuicidal self-injury. SE—standard error, *t*-Value—*t*-tests, df—degrees of freedom, *p*—level of significance. * *p* < 0.05. ** *p* < 0.01.
